# Identification, expression and interaction analyses of calcium-dependent protein kinase (CPK) genes in canola (*Brassica napus* L.)

**DOI:** 10.1186/1471-2164-15-211

**Published:** 2014-03-19

**Authors:** Hanfeng Zhang, Wu-Zhen Liu, Yupeng Zhang, Min Deng, Fangfang Niu, Bo Yang, Xiaoling Wang, Boya Wang, Wanwan Liang, Michael K Deyholos, Yuan-Qing Jiang

**Affiliations:** 1 State Key Laboratory of Crop Stress Biology for Arid Areas and College of Life Sciences, Northwest A & F University, Yangling, Shaanxi 712100, China; 2 Department of Biological Sciences, University of Alberta, Edmonton, Alberta T6G 2E9, Canada

**Keywords:** Abiotic stress, Basic leucine zipper transcription factor, *Brassica napus*, Calcium-dependent protein kinase, Protein phosphatase 2C

## Abstract

**Background:**

Canola (*Brassica napus* L.) is one of the most important oil-producing crops in China and worldwide. The yield and quality of canola is frequently threatened by environmental stresses including drought, cold and high salinity. Calcium is a well-known ubiquitous intracellular secondary messenger in plants. Calcium-dependent protein kinases (CPKs) are Ser/Thr protein kinases found only in plants and some protozoans. CPKs are Ca^2+^ sensors that have both Ca^2+^ sensing function and kinase activity within a single protein and play crucial roles in plant development and responses to various environmental stresses.

**Results:**

In this study, we mined the available expressed sequence tags (ESTs) of *B. napus* and identified a total of 25 CPK genes, among which cDNA sequences of 23 genes were successfully cloned from a double haploid cultivar of canola. Phylogenetic analysis demonstrated that they could be clustered into four subgroups. The subcellular localization of five selected BnaCPKs was determined using green fluorescence protein (GFP) as the reporter. Furthermore, the expression levels of 21 BnaCPK genes in response to salt, drought, cold, heat, abscisic acid (ABA), low potassium (LK) and oxidative stress were studied by quantitative RT-PCR and were found to respond to multiple stimuli, suggesting that canola CPKs may be convergence points of different signaling pathways. We also identified and cloned five and eight Clade A basic leucine zipper (bZIP) and protein phosphatase type 2C (PP2C) genes from canola and, using yeast two-hybrid and bimolecular fluorescence complementation (BiFC), determined the interaction between individual BnaCPKs and BnabZIPs or BnaPP2Cs (Clade A). We identified novel, interesting interaction partners for some of the BnaCPK proteins.

**Conclusion:**

We present the sequences and characterization of CPK gene family members in canola for the first time. This work provides a foundation for further crop improvement and improved understanding of signal transduction in plants.

## Background

Plants have developed complex signal transduction pathways to cope with fluctuating environmental changes throughout their life cycles. Environmental stresses, such as high salinity, drought, cold and heat affect not only crop yield but also quality. Ca^2+^ is a ubiquitous second messenger that is involved in the signaling of a variety of environmental and developmental stimuli. In response to these stimuli, cells generate transient changes in the intracellular Ca^2+^ concentration and these changes are sensed and decoded by Ca^2+^ sensors including calmodulins (CaMs), calmodulin-like proteins (CMLs), calcineurin B-like proteins (CBLs) and calcium-dependent protein kinases (CDPKs/CPKs) [1,2]. The CPKs constitute one of the largest calcium sensing subfamilies of serine/threonine protein kinases that have been identified throughout the plant kingdom from algae to angiosperms and also in some protozoans [3,4].

CPKs contain four characteristic functional domains including an N-terminal domain, a protein kinase domain, an autoinhibitory domain and a calmodulin-like domain [5-7]. The N-terminal domain often contains myristoylation or palmitoylation sites for membrane association [5] and is highly variable, which may determine its substrate specificity [8,9]. The C-terminal calmodulin-like domain (regulatory domain) contains EF-hands for binding to Ca^2+^[10]. Previous studies identified a total of 34 CPK genes in the model plant Arabidopsis [5,7]. An investigation of the sequenced rice genome revealed 31 CPK genes in rice [11-13]. A similar attempt in bread wheat identified 20 CPK genes [14]. Phylogenetic analysis of CPKs from both Arabidopsis and rice demonstrated that they can be clustered into four distinct subgroups [15].

In Arabidopsis, rice, and a few other plant species, functional analyses of CPKs using gain-of-function and loss-of-function mutants have revealed the biological function of CPKs in an array of physiological processes, including accumulation of storage starch in immature seeds of rice [16], pollen tube elongation [17], root development [18], cell division, differentiation and programmed cell death [19,20] and more importantly, in abiotic and biotic stress and hormone signaling [1,15,21]. For instance, members of CPK families in Arabidopsis and rice have been reported to be involved in drought [22-25], salt [22-24,26,27], cold [23,28,29], abscisic acid (ABA) [30-32], reactive oxygen species (ROS) [33,34] signaling pathways and immune responses against pathogens [33,35,36]. Besides, in ABA signaling, three Arabidopsis CPK proteins, CPK4, CPK11 and CPK32, have been found to phosphorylate ABA-responsive bZIP-type transcription factors (ABFs/AREBs) [30,32], though the exact phosphorylation sites have not been determined. In addition, two tobacco (*Nicotiana tabacum*) CPDK genes, *NtCDPK2* and *NtCDPK3* were identified to play an essential role in defense response and, *NtCDPK2* functions together with stress-induced MAPKs (mitogen-activated protein kinases) to control response specificity to abiotic and biotic stress [37,38]. Taken together, the above reports clearly demonstrate that plant CPK genes are essential to confer increased resistance to abiotic and biotic stresses. However, the specific functions of many CPKs and their target proteins in plants are still not clear.

Despite extensive studies of CPKs in many other species [5,7,11-14,39,40], little is known about this gene family in canola (*Brassica napus* L.). Until now, only one CPK (partial sequence) from *B. napus* has been deposited in GenBank (accession number DQ071551.1) and the role of CPK in canola growth and development as well as in response to abiotic and biotic stresses have not been reported at all. Canola, with low erucic acid and glucosinolates, is one of the most important oil crops in China and worldwide. Losses from adverse environmental conditions greatly influence canola production and quality. To understand the molecular mechanisms of canola responses to abiotic stresses is a prerequisite for many approaches towards improving stress tolerance to meet increasing demands for edible oil. This is a promising approach to improve stress tolerance of plants by modulating the expression of key genes in plant breeding. Canola is an allotetraploid, and its complete genome sequence is not publicly available yet.

In view of the importance of CPK genes in biotic and abiotic stress responses, we initiated a project to isolate CPKs from canola. In the present study, we systematically identified and cloned the cDNA sequences of 23 CPK genes from canola by mining the expressed sequence tag database. Evolutionary history reconstruction via comparison with CPK genes from other plant species was performed. Furthermore, we analyzed the subcellular localization of canola CPKs, and their expression profiles under abiotic stress, which suggested that they participated in stress-related signal transduction pathways. Furthermore, interaction of canola CPK with Clade A bZIPs and PP2Cs were examined through yeast two-hybrid assays and BiFC. The current work represents the first comprehensive study of CPK genes in canola and provides a solid foundation for further functional investigation of this important gene family in canola.

## Results and discussion

### Identification and cDNA cloning of calcium-dependent protein kinase genes in canola

Ca^2+^-dependent protein kinases (CPKs) are both Ca^2+^ sensors and Ser/Thr kinases that are found only in plants and some protozoans. Because sequences or functions of CPK genes in oilseed rape (canola, *B. napus*) have not been reported, we were motivated to characterize this important gene family in canola. As the first step to understand the roles of CPK genes in canola growth and development, as well as in response to abiotic and biotic stress conditions, we initiated a project to identify and clone the cDNA sequences of CPK genes from canola. Considering the high similarity between Arabidopsis and canola genomes and the fact that sequencing of canola genome has not been finished, we used the cDNA sequences of 34 Arabidopsis CPK genes as the queries in BLAST searches of the expressed sequence tag (EST) database of *B. napus* in NCBI (http://www.ncbi.nim.nih.gov/dbEST.html) with an E-value cut-off of lower than 10^-4^. The obtained ESTs were further examined to remove vector sequences through VecScreen (http://www.ncbi.nlm.nih.gov/tools/vecscreen/) and were assembled to obtain contigs and singlets, which were then reciprocally BLAST searched against the Arabidopsis database (http://www.arabidopsis.org/Blast/index.jsp) to identify putative orthologs in the model plant Arabidopsis. As a result, we identified 336 ESTs representing 25 canola *CPK*s (Table 1, Additional file 1: Table S1). These ESTs were clustered into 38 singlets and 57 contigs (data not shown). For comparison and clarity, the nomenclature of respective canola CPK genes was annotated according to the Arabidopsis orthologs with *Bna* abbreviating *B. napus* (Table 1). We noted that among the 25 *BnaCPKs* annotated, *BnaCPK24* had the largest number (47) of ESTs, followed by *BnaCPK3* with a total of 38 ESTs and, *BnaCPK8* with 32 ESTs while *BnaCPK10, -16* and *-18* had only one EST each (Additional file 1: Table S1).

**Table 1:** **Calcium-dependent protein kinase (CPK) genes identified and cloned from canola (*Brassica napus *** **L.)**

**Gene**	**GenBank acc no.**	**EST count**	**Arabidopsis homolog/AGI no.**	**Rice homolog/locus**^**a**^	**CDS (bp)**	**Protein M.W. (kDa)**	**pI**	**No. of EF-hands**^**b**^	**N-termianal AA.**	**Palmitoylation sites**^**c**^	**Myristoylation sites**^**d**^
*BnaCPK1*	JX122905	7	*AtCPK1/At5g04870*	*OsCPK10/LOC_Os03g57450.1*	1788	66.3	5.11	4	MGNTCVGP	Yes	n.d.
*BnaCPK2*	JX122906	4	*AtCPK2/At3g10660*	*OsCPK10/LOC_Os03g57450.1*	1854	68.8	5.27	4	MGNVCVGP	Yes	n.d.
*BnaCPK3*	JX122910	38	*AtCPK3/At4g23650*	*OsCPK1/LOC_Os01g43410.1*	1578	58.7	6.31	4	MGHRHSKS	n.d.	Yes
*BnaCPK4*	KF169735	2	*AtCPK4/At4g09570*	*OsCPK24/LOC_Os11g07040.1*	1503	56.3	5.12	4	MEKPSSRR	n.d.	n.d.
*BnaCPK5*	JX122911	30	*AtCPK5/At4g35310*	*OsCPK13/LOC_Os04g49510.1*	1638	61.3	5.47	4	MGNSCRRS	Yes	n.d.
*BnaCPK6*	JX122912	14	*AtCPK6/At2g17290*	*OsCPK13/LOC_Os04g49510.1*	1647	61.5	5.14	4	MGNSCRGS	Yes	n.d.
*BnaCPK7*	JX122913	15	*AtCPK7/At5g12480*	*OsCPK20/LOC_Os07g38120.1*	1599	59.5	5.97	4	MGNCCGSP	Yes	n.d.
*BnaCPK8*	JX122914	32	*AtCPK8/At5g19450*	*OsCPK20/LOC_Os07g38120.1*	1605	60.2	6.22	4	MGNCCASP	Yes	n.d.
*BnaCPK9*	JX122915	11	*AtCPK9/At3g20410*	*OsCPK19/LOC_Os07g33110.1*	1620	60.5	5.92	4	MGNCFAKN	Yes	Yes
*BnaCPK10*	JX122900	1	*AtCPK10/At1g18890*	*OsCPK9/LOC_Os03g48270.1*	1632	61.4	6.44	4	MGNCNVCV	Yes	n.d.
*BnaCPK11*	JX122901	9	*AtCPK11/At1g35670*	*OsCPK24/LOC_Os11g07040.1*	1494	56.1	5.17	4	MEKANPRR	n.d.	n.d.
*BnaCPK12*	JX122902	26	*AtCPK12/At5g23580*	*OsCPK24/LOC_Os11g07040.1*	1464	55.8	5.34	4	MANKSRTR	n.d.	n.d.
*BnaCPK13*	JX122903	24	*AtCPK13/At3g51850*	*OsCPK3/LOC_Os01g61590.1*	1587	59.5	6.49	4	MGNCCRSP	Yes	n.d.
*BnaCPK15*	JX122904	10	*AtCPK15/At4g21940*	*OsCPK19/LOC_Os07g33110.1*	1710	64.4	6.09	4	MGCFSSKH	Yes	Yes
*BnaCPK17*	KF169737	22	*AtCPK17/At5g12180*	*OsCPK2/LOC_Os01g59360.1*	1572	58.1	5.81	4	MGNCCSGR	Yes	Yes
*BnaCPK18*	KF169738	1	*AtCPK18/At4g36070*	*OsCPK18/LOC_Os07g22710.1*	1626	61.5	7.47	4	MGLCFSSP	Yes	Yes
*BnaCPK21*	JX122907	4	*AtCPK21/At4g04720*	*OsCPK19/LOC_Os07g33110.1*	1578	59.2	6.45	4	MGCLSSKH	Yes	Yes
*BnaCPK24*	KF169739	47	*AtCPK24/At2g31500*	*OsCPK29/LOC_Os12g12860.1*	1833	69.3	6.8	4	MGSCVSSP	Yes	Yes
*BnaCPK28*	JX122909	11	*AtCPK28/At5g66210*	*OsCPK18/LOC_Os07g22710.1*	1623	61	8.65	4	MGLCFSAI	Yes	Yes
*BnaCPK29*	JX122908	4	*AtCPK29/At1g76040*	*OsCPK19/LOC_Os07g33110.1*	1617	61	6.17	4	MDTRTKHF	n.d.	n.d.
*BnaCPK30*	KF169740	4	*AtCPK30/At1g74740*	*OsCPK9/LOC_Os03g48270.1*	1641	61.9	6.62	4	MGNCIACV	Yes	Yes
*BnaCPK32*	KF169741	4	*AtCPK32/At3g57530*	*OsCPK20/LOC_Os07g38120.1*	1617	60.9	6.17	4	MGNCCGTA	Yes	Yes
*BnaCPK34*	KC414030	10	*AtCPK34/At5g19360*	*OsCPK2/LOC_Os01g59360.1*	1458	54	5.28	4	MGNCCGRD	Yes	n.d.

^a^InParanoid, http://inparanoid.sbc.su.se/cgi-bin/index.cgi. NA, InParanoid could not detect any orthologs for this gene.

^b^*The number of EF-hands were predicted by SMART scan and by sequence alignment.*

^c^*The palmitoylation site were predicted by CSS-Palm 3.0.*

^d^*The myristoylation sites were predicted by Myristoylator.*

n.d., not detected.

To facilitate the subsequent phylogenetic, GFP fusion, yeast two-hybrid and other analyses, we designed primers based on the identified ESTs for each of the *BnaCPK* genes to allow us to amplify the full-length cDNA sequences, employing both regular RT-PCR and RACE technologies. As a result, we succeeded in cloning the cDNA sequences of 23 of the 25 *BnaCPK* genes (Table 1). For seven BnaCPK genes, two slightly different alleles were cloned, which were *BnaCK2, -3, -4, -5, -8, -10* and *-13.* For comparison, we also identified putative orthologs of these *BnaCPK* genes in both Arabidopsis and rice (Table 1).

### Sequence analysis of canola BnaCPK genes

After examining the sequences of the 23 cloned *BnaCPK* genes, we found that the length of their coding regions (CDS) ranged from 1458 to 1854 bp. The full-length canola CDPK proteins conceptually translated from the full-length cDNA we cloned were 485 to 617 amino acids long. The molecular weight of the predicted BnaCPK proteins range from 54 to 69.3 kDa, with an isoelectric point (pI) between 5.11 and 8.65 (Table 1). We also found that the cDNA sequence identity of different *BnaCPKs* ranged from 49.8% to 86.5% (50.4-86.5% similarity), whereas the amino acid sequence identity of different BnaCPKs ranged from 33.1% to 94.2% (51.8-97% similarity, Additional file 2: Table S2).

It was noted that the deduced amino acid sequences of the 23 BnaCPK genes showed high conservation both in size and structure, with a kinase domain near the N-terminus and four Ca^2+^-binding EF-hand motifs at the C-terminus (Additional file 3: Figure S1). We found that there is an insert of 38 and 26 amino acids in the kinase domain of BnaCPK1 and -24, respectively (Additional file 3: Figure S1). As Ca^2+^ sensors, CPK proteins are known to be able to bind Ca^2+^ ions through the EF-hand motifs. Indeed, all the 23 canola CPK proteins with full-length sequences had a calmodulin-like structure with four EF-hand motifs at their C-terminus (Table 1, Additional file 3: Figure S1). We did not observe any of the 23 BnaCDPKs with fewer than four EF-hands as reported in Arabidopsis, rice and maize [5,11,41].

As a special class of post-translational modifications (PTMs), many proteins including CPKs could be covalently modified by a variety of lipids, such as myristate (C14) and palmitate (C16) [42]. In rice, it has been shown that OsCPK19(OsCDPK2) is both myristoylated and palmitoylated and targeted to the membrane fraction. Both modifications of OsCPK19 are required, myristoylation being essential for membrane localization and palmitoylation for its full association [43]. Another study of tobacco NtCDPK2 and NtCDPK3 also demonstrated that myristoylation and palmitoylation–mediated membrane localization is necessary for proper function of these two kinases in regulating stress response [9]. Although most of lipid modifications are irreversible, protein S-palmitoylation, also called as thioacylation or S-acylation, could reversibly attach 16-carbon saturated fatty acids to specific cysteine residues in protein substrates through thioester linkages [44,45]. Palmitoylation will enhance the surface hydrophobicity and membrane affinity of protein substrates, and play important roles in modulating proteins’ trafficking, stability, and sorting, etc [42,44,45]. Our bioinformatic analysis revealed that ten of the 23 BnaCPK proteins bear potential N-myristoylation motifs (MGXXXS/T) for membrane association and they are BnaCPK3, -9, -15, -17, -18, -21, -24, -28, -30 and -32 (Table 1). We also found that N-terminal sequences of 18 of the 23 BnaCPK proteins, except BnaCPK3, -4, -11, -12 and -29, harbored palmitoylation sites (Table 1, Additional file 3: Figure S1), which indicated that myristoylation and palmitoylation modifications of BnaCPK might be functional in membrane targeting and stability of BnaCPK proteins.

### Phylogenetic analysis of CDPK gene family

To better understand the evolutionary history of the CDPK/CPK gene family, we used a HMM-based search to identify and retrieve CPK genes from a variety of species, especially those with sequenced genomes. We mined the genomes of those plant species that represent the most important milestones characterizing the evolution of land plants (embryophytes). As a result, we identified 29 distinct *CPKs* from *B. distachyon*, 30 from *S. bicolor*, 11 from *S. moellendorffii*, 26 from the moss *P. patens,* 15 from the model alga *C. reinhardtii*, three from *O. tauri* and four from *O. lucimarinus* (Additional file 4: Table S3). Similarly, we mined the recently sequenced Chinese cabbage (*Brassica rapa* L.) genome, a progenitor of *B. napus* and identified a total of 51 *BraCPK* genes whose translated protein sequences harbored typical domains and motifs of CPK proteins including the N-terminal kinase domain and C-terminal EF-hand motif. Further, an orthologous comparison of these 51 *BraCPK* genes to the 34 *AtCPK* genes indicated that only 29 pairs of orthologs exist, with the remaining being alleles of these 29 genes (Additional file 5: Table S4), suggesting a genome duplication event may have caused the expansion of some genes in *B. rapa*. We then compared the 51 *BraCPK* genes to the 23 *BnaCPK* genes we cloned and, confirmed that for all the 23 *BnaCPKs*, orthologs could be identified (Additional file 5: Table S4). The identification of multiple members of CPK gene families in all the analyzed plant species suggested that CPK proteins may play a role in response to developmental and environmental stimuli.

It has been observed that the size of the canola CPK gene family is comparable to that of either Arabidopsis or rice. On the other hand, only six, four and 15 CPK genes were identified from the three lower organisms *O. tauri*, *O. lucimarinus* and *C. reinhardtii*, respectively (Additional file 4: Table S3). Furthermore, we only identified 11 CPKs genes from the lycophyte *S. moellendorffii*. This indicates an expansion of CPK gene family after the divergence of flowering plants from the remainder of the tracheophyte lineage. Furthermore, comparing the numbers of CPK genes between lower and higher organisms indicates an obvious expansion of this gene family during the long history of evolution, which is supported by a very recent study [46].

**Figure 1:**
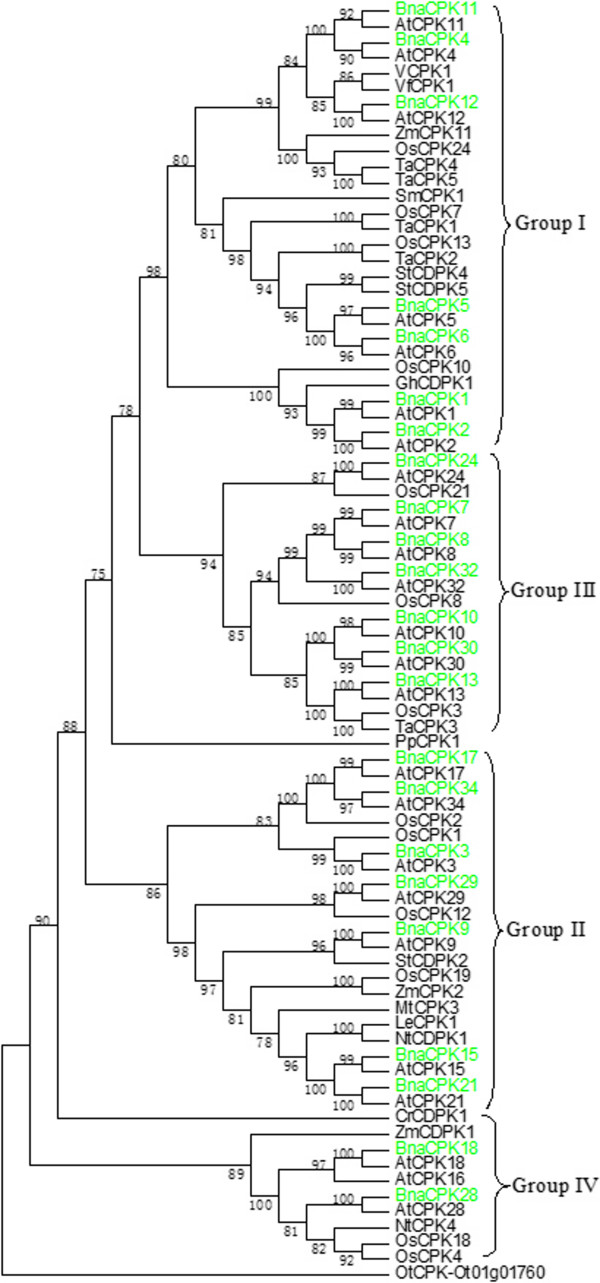
**Phylogenetic relationship of canola CPK proteins with CPKs from other species.** Protein sequences were aligned using ClustalX (v1.83) and a maximum parsimony (MP) bootstrap consensus tree was drawn using MEGA5.1. The CPKs can be clustered into four major groups (I-IV). The percentage of replicate trees is shown on the branches and it is calculated in the bootstrap test (1000 replicates) for the associated taxa being clustered together. The 23 BnaCPKs reported in this study are highlighted in green. The tree was rooted with a CDPK identified from the smallest free-living organism, *Ostreococcus tauri.* At, *Arabidopsis thaliana*; Bna, *Brassica napus*; Cr, *Chlamydomonas reinhardtii*; Gh, *Gossypium hirsutum*; Le, *Lycopersicon esculentum*; Mt, *Medicago truncatula*; Nt, *Nicotiana tabacum*; Os, *Oryza sativa*; Pp, *Physcomitrella patens*; Sb, *Sorghum bicolor*; Sm, *Selaginella moellendorffii;* St, *Solanum tuberosum*; Ta, *Triticum aestivum*; Vf, *Vicia faba*; Zm, *Zea mays*, Ol, *Ostreococcus lucimarinus*; Ot, *Ostreococcus tauri.*

The amino acid sequences of BnaCPKs together with CPKs from other species were aligned and a consensus maximum parsimony dendrogram was produced (Figure 1, Additional file 6: Figure S2). As shown by the tree’s topology, the CPK proteins from various species could be divided into four distinct groups (I to IV), each supported by highly significant bootstrap values, as reported previously with rice and wheat CPK proteins [11,14]. The 23 canola CPKs were distributed in each of the four groups, with BnaCPK1, -2, -4, -5, -6, -11 and -12 belonging to Group I, BnaCPK3, -9, -15, -17, -21, -29 and -34 belonging to Group II, BnaCPK7, -8, -10, -13, -24, -30 and -34 belonging to Group III, and BnaCPK18 and -28 Group IV (Figure 1, Additional file 6: Figure S2). We also reconstructed the phylogenetic tree of CPK proteins from Arabidopsis, canola, *B. rapa* and rice, and also found that the CPKs could be clustered into four distinct groups (Additional file 6: Figure S2). It was also noted that the 23 BnaCPK members were always closely clustered in the same group as AtCPK and *BraCPK* orthologs, as expected for these two members of the family Brassicaceae. In addition, phylogenetic analysis based on predicted amino acid sequences could distinguish 11 closely related pairs of canola CPKs: BnaCPK1/2 (76.2% identity), BnaCPK4/11 (94.2% identity), BnaCPK5/6 (88.5% identity), BnaCPK7/8 (88.1% identity), BnaCPK10/30 (84.9% identity), BnaCPK15/21 (76.2% identity), BnaCPK17/34 (85.3% identity) and BnaCPK18/28 (71.2% identity) (Additional file 2: Table S2; Additional file 6: Figure S2). Past research has demonstrated that closely related pairs of CPKs in Arabidopsis may play similar roles or are co-localized in certain subcellular compartments. For instance, In Arabidopsis, CPK4 and CPK11 regulate ABA signaling [32], CPK17 and CPK34 increase pollen tube tip growth [17], and CPK7 and CPK8 are both localized in the plasma membrane [47]. Knowledge of the phylogenetic relationships of canola CPKs should facilitate dissection of their function.

To gain insights into the evolutionary history of the CPK family in plants, we used the deduced amino acid sequences of 23 canola CPK sequences as well as those identified from a variety of representative plant species to infer the phylogenetic relationships (Additional file 4: Table S3; Additional file 6: Figure S2). The resulting dendrogram showed most CPKs, especially those from land plants could essentially be classified into four distinct groups (I to IV), based on to their sequence similarity, which was further supported by the high bootstrap values. However, some of the CPKs mainly from green algae, plus a few from the pteridophyte *S. moellendorffii*, the bryophyte *P. patens*, *S. bicolor* and *B. distachyon* constitute a distinct clade, which is similar to a recent analysis [46].

From our phylogenetic analysis, multiple alignment and domain analysis of BnaCPKs in canola, we concluded some of the CPK family members may be conserved among monocots while others were lost after the divergence of the monocots and dicots. The phylogenetic analysis together with the domain motif analysis presented here will facilitate the functional annotation and study of canola CPKs.

### Subcellular localization of selected BnaCPK proteins

**Figure 2:**
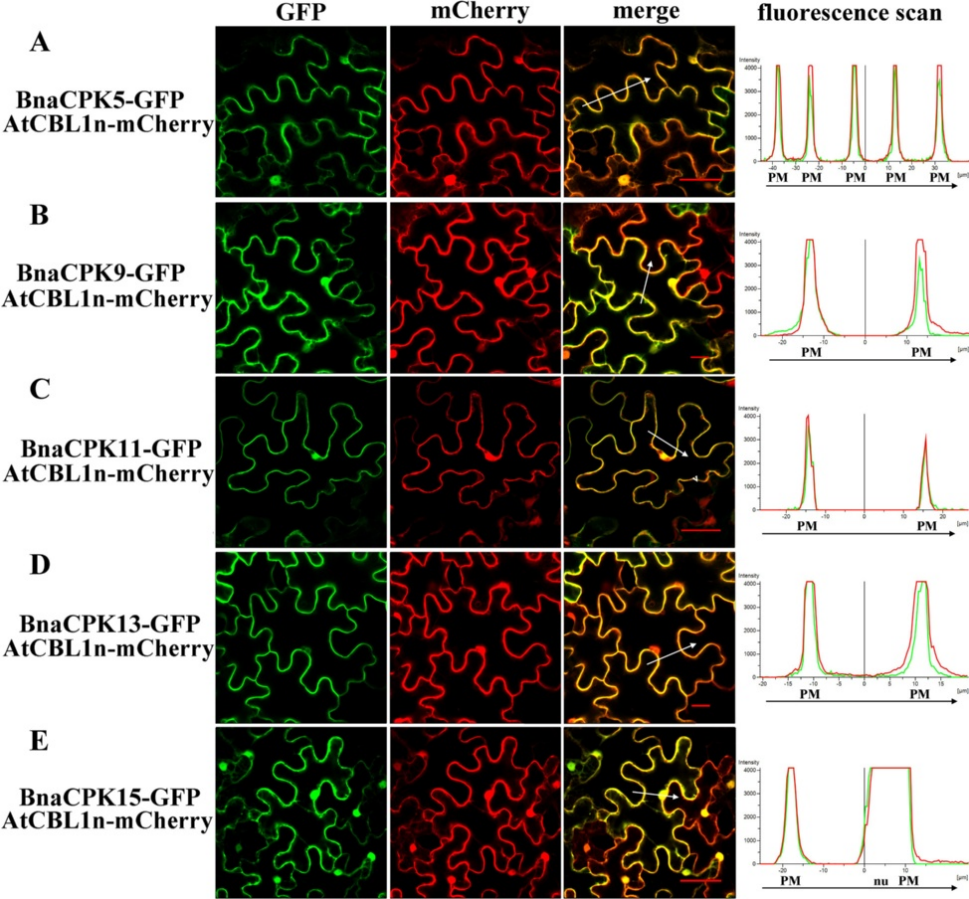
**Subcellular localization of selected BnaCPK-GFP fusion proteins in intact *N. benthamiana *leaf epidermal cells and protoplasts.** Panels **A**-**E** represent the subcellular localizations of BnaCPK5-GFP, BnaCPK9-GFP, BnaCPK11-GFP, BnaCPK13-GFP and BnaCPK15-GFP, together with a plasma marker AtCBL1n fused with mCherry, respectively. A white arrow marks the region and direction in which the distribution of fluorescence intensities was scanned. The left panel is GFP fluorescence, the middle mCherry field and the right represents an overlay of the two images. PM, plasma membrane; nu, nuclei. Bar = 50 μm.

To examine the subcellular localizations of canola CPKs, we used translational fusions with green fluorescence protein (GFP). In total, six canola CPKs (BnaCPK3, -5, -9, -11, -13, -15) were tested regarding their subcellular localization in *N. benthamiana* leaf cells. Of these, constructs with five of the selected BnaCPK-GFP genes produced detectable fluorescence (Figure 2). Fusion with BnaCPK3 did not. Among the CPKs tested, BnaCPK3, -9 and -15 were predicted to have a myristoylation motif, while BnaCPK3, -5, -9, -13 and -15 had palmitoylation sites (Table 1). We also expressed each of the *BnaCPK-GFP* fusion genes together with a putative plasma membrane marker AtCBL1n [48] fused to the *mCherry* reporter gene. The GFP fusion proteins of BnaCPK9 and -15 were found to be associated with plasma membranes and nuclei (Figure 2B and E). However, it seemed that proteins lacking a myristoylation and/or palmitoylation motif were also localized to the plasma membrane since three canola CPKs, BnaCPK5, -11 and -13 displayed fluorescence at the plasma membrane and nuclei (Figure 2A, C and D). This may indicate the existence of additional motifs that provide these CPKs with their membrane association ability, which probably was not detected by the computer program. Similar observations were made with wheat CPK3 and -15 proteins, which lacked myristoylation motifs but were associated with membranes [14]. We also found that all five BnaCPKs (BnaCPK5, -9, -11, -13 and -15) were localized in the nucleus as well (Figure 2).

As a control, we tested the subcellular localization of GFP or mCherry protein alone in the leaf cells of *N. benthamiana* and observed that the signal was spread throughout the cytoplasm of the leaf epidermal cells as well as in the nuclei (data not shown).

### Expression profiles of CDPK genes in response to abiotic stress and hormone treatments

**Figure 3:**
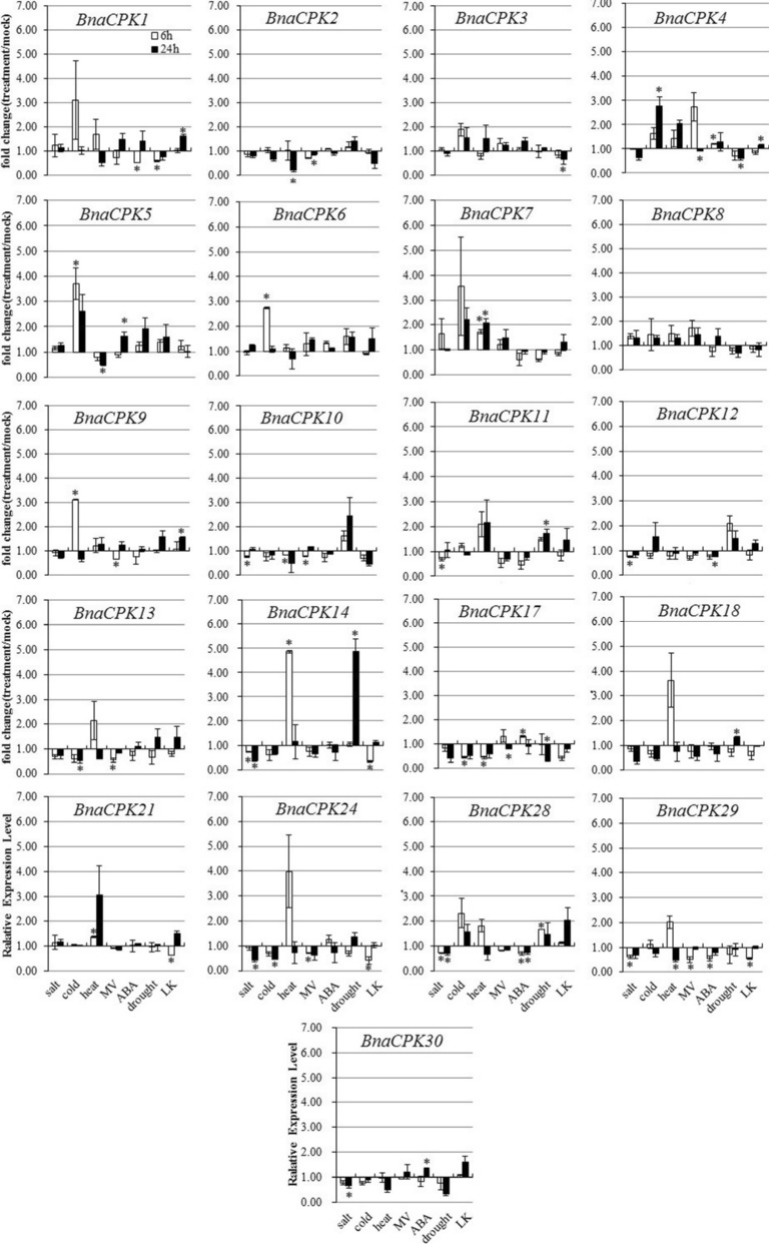
**Expression analyses of canola CPK genes in response to a variety of treatments, including salt (200 mM NaCl), cold (4°C), heat (37°C), 10 μM Paraquat (Methyl viologen, MV), 50 μM ABA, drought and low potassium (LK).** Data is the mean (treatment/mock, linear scale) of three biological replicates ± S.E. Asterisks denote significant differences between treated and mock plants by Student *t*-test (*p* ≤ 0.05).

One of the most important functions of CPK proteins is to sense changes in cytosolic calcium concentration during biotic and abiotic challenges [15,21]. To understand the responses of CPK genes to environmental stresses and hormone stimuli in canola, we used quantitative RT-PCR (qRT-PCR) to analyze the transcript changes of canola CPK genes. 18-day-old canola seedling tissues of three biological replicates were collected after being exposed to salt, cold, heat, drought, Paraquat (methyl viologen, MV), ABA, and low potassium (LK) for 6 and 24 h. We chose these abiotic stresses and hormone application for two reasons, since salt, cold, heat and drought are the most devastating factors influencing crop yielding and quality and, ABA is a stress hormone playing very important roles in plant growth, development and responses to abiotic stresses including salinity, drought and to a less extent cold. ROS acts as signaling molecules in stress adaptation, hormone signaling, and programmed cell death (PCD) [49]. Past reports have demonstrated that a few CPK genes in Arabidopsis, rice and potato are involved in either drought [22,25], salinity [23,24,26,27], cold [23,29], ABA [30-32,50] or ROS [26,34] signaling. Potassium (K^+^) is the most important and abundant cation in living plant cells. However, due to the limited availability of K^+^ in soils, many crops often suffer from K^+^-deficiency stress (LK). Although the CBL-CIPK system has been shown to play an important role in LK responses through phosphorylating K^+^ channel-AKT1 [51,52], the role of CPK genes in LK responses is unknown. Emerging evidence also suggests that ROS participate in LK responses [53]. Therefore, we measured changes in transcript abundance of 21 *BnaCPK* genes in response to the seven stress treatments described above (Figure 3). The expression profiles of the remaining four genes were not examined because the specificity of the designed primers was insufficient.

Statistical analysis of three biological replicates revealed that transcript abundance of 20 out of 21 canola CPK genes, except *BnaCPK8*, showed significant changes in response to at least one stress treatment (Figure 3). Often, one *CPK* was able to respond to several treatments and, on the other hand, one treatment affected multiple CPK genes. For example, eight genes (*BnaCPK10, -11, -12, -14, -24, -28, -29* and *-30*) responded to saline stress and they were all down regulated (Figure 3). Cold stress significantly induced *BnaCPK4, -5, -6, -9* but repressed *BnaCPK13, -17, -24*. In contrast, the transcript abundance of *BnaCPK7, -14* and *-21* was up-regulated by heat stress, while *BnaCPK2, 5, 10, 17* and *29* down-regulated. MV-induced oxidative stress increased the transcript accumulation of *BnaCPK5*, but decreased that of *BnaCPK2, -4, -9, -10, -13, -17, -24* and *-29.* When 50 μM ABA was applied to soil-grown canola seedlings, it induced the expression of *BnaCPK 4, -17* and *-30* while repressed *BnaCPK1, -12, -28* and *-29*. In response to drought treatment, *BnaCPK11, -14, -18* and *-28* were induced, whereas *BnaCPK1, -4* and *-17* repressed. Finally, we assayed the transcript levels of 21 BnaCPK genes in hydroponically-grown canola roots when subjected to low K^+^ treatment, and found that *BnaCPK1, -4* and *-9* were up-regulated by LK compared to mock treatment, while *BnaCPK3*, -14, -21, -24 and -29 were down-regulated at early or late time-points. Taken together, our data not only demonstrated that *BnaCPK* genes are involved in salt, cold, heat, MV, ABA, drought stresses, as reported with *CPKs* in Arabidopsis, rice and a few other species, but also showed that *BnaCPKs* likely respond to Ca^2+^ fluctuation caused by LK stress. From another point of view, among these 20 BnaCPK genes analyzed, *BnaCPK4* also responded to cold, MV, ABA, drought and LK treatments and, *BnaCPK5* responded to cold, heat and MV treatments. On the other hand, *BnaCPK29* was down-regulated by salt, heat, MV, ABA and LK treatments (Figure 3). Therefore, canola CPKs seemed to be mediate cross-talk among different signaling pathways.

Taken together, analysis of transcript expression patterns of 21 BnaCPK genes in canola seedlings after various stress treatments suggested that different CPK genes may participate in the signaling process to a single stress and a single CPK likely plays a role in multiple stress responses.

### Cloning and sequence analysis of Clade A bZIP and PP2C genes in canola

We sought to identify the substrates of BnaCPK proteins, with the knowledge that multiple BnaCPK genes responded to different abiotic stress treatments as assayed by qRT-PCR and that ABA is a phytohormone that participates in many abiotic stress signaling pathways [54]. Since ABA responsive cis-element [55] binding factor/protein (ABF/AREB) transcription factors (TFs) in the Clade A basic leucine zipper (bZIP) family regulate ABRE-dependent gene expression [56] and, previous studies demonstrated that Arabidopsis CPK4 and CPK11 exert their functions in ABA signaling through phosphorylating ABF transcription factors ABF1 and ABF4/AREB2, while CPK32 phosphorylates ABF4 and interacts with ABF1, ABF2/AREB1 and ABF3 as well [30,32,34]. In addition, AtCPK10 and AtCPK30 are known to interact with ABF4 in yeast two-hybrid assays [30]. However, these interactions have not previously been demonstrated *in vivo*.

To test whether BnaCPKs could similarly act upstream of Clade A bZIP TFs, we first used cDNA sequences of Arabidopsis ABF1, -2, -3, -4, AREB3 and ABI5 genes to obtain canola ESTs that showed significant homology to five of these six genes. These were *BnaABF1, BnaABF3, BnaABF4, BnaAREB3, BnaABI5* (Additional file 7: Table S5). We did not identify any ESTs encoding BnaABF2. We further cloned the full-length cDNA sequences of all five canola TFs (Table 2). Sequence and domain analysis as well as phylogenetic comparison of the translated protein sequences revealed that these five bZIP proteins showed very high identity/similarity to their orthologs in Arabidopsis and also harbored characteristic domains and motifs (Additional file 8: Figure S3).

**Table 2:** **Clade A bZIP and PP2C genes identified and cloned from Canola (*Brassica napus *** **L.)**

**Gene**	**GenBank acc no.**	**EST count**	**Arabidopsis ortholog/AGI no.**^**a**^	**length of CDS (bp)**	**Protein M.W. (kDa)**	**pI**
*BnaABF1*	JX122892	6	ABF1/At1g49720	1089	39.8	9.137
*BnaABF3*	JX122893	23	ABF3/At4g34000	1233	45.5	8.699
*BnaABF4*	JX122894	23	ABF4/At3g19290	1188	43.1	9.639
*BnaAREB3*	JX122898	21	AREB3/At3g56850	849	31.4	9.245
*BnaABI5*	KC414029	16	ABI5/At2g36270	1317	46.2	8.96
*BnaABI1*	JX122895	62	ABI1/At4g26080	1263	46.2	6.696
*BnaABI2*	JX122896	5	ABI2/At5g57050	1269	46.4	6.093
*BnaHAB1*	JX122916	36	HAB1/At1g72770	1455	52.6	4.765
*BnaHAB2*	JX122917	10	HAB2/At1g17550	1539	56.1	4.587
*BnaAHG1*	KF365484	13	AHG1/At5g51760	1257	45.5	6.101
*BnaAHG3*	JX122897	86	AHG3/At3g11410	1206	43.3	6.426
*BnaHAI1*	N/A	7	HAI1/At5g59220			
*BnaHAI2*	KF365485	8	HAI2/At1g07430	1311	48.9	6.741
*BnaHAI3*	KF365487	7	HAI3/At2g29380	1101	40.9	5.902

^a^The gene synteny was detected by InParanoid (http://inparanoid.sbc.su.se/cgi-bin/index.cgi).

N/A. cDNA cloning was not successful.

In contrast to kinases, protein phosphatases act to remove the phosphorous group from the phosphorylated target therefore negatively regulating signaling process. Over half of all Arabidopsis protein phosphatases belong to the monomeric PP2C subfamily, which has 76 members, belonging to ten or more subgroups or clades [57]. During the long history of evolution from prokaryotes to multicellular eukaryotes, PP2Cs have expanded and diversified, with an increase in the number of distinct clades and in the total number of genes encoding for PP2Cs [57]. For instance, Clade A PP2Cs of plants are not found in prokaryotes or in non-plant eukaryotes, such as yeast. In Arabidopsis, Clade A PP2C are highly correlated with response to ABA and drought stress and, molecular studies have identified interactions with proteins including transcription factors of the homeodomain-leucine zipper (HD-ZIP) class, a chromatin remodeling factor, and members of the SnRK (sucrose non-fermenting related kinase) and MAPK (mitogen-activated protein kinase) families, or PYR/PYL/RCAR class of ABA receptors [57]. More recently, direct interactions between Arabidopsis CPK11 and members of clade A PP2Cs were reported [58], suggesting that CPK-PP2C interaction may be a common mechanism in plant signaling under developmental stimuli and/or (a)biotic stresses. However, a detailed and systematical examination of interactions between CPKs and Clade A PP2C or CPKs and ABF/AREB/ABI5 clade is lacking. Besides, the sequences and function of these PP2C and bZIP TFs in canola has not been reported.

To this end, we also identified, cloned and sequenced the cDNA sequences of clade A PP2Cs in canola, using a technique similar to *BnaCPK* cloning as described previously. We first identified the ESTs for these PP2C genes (Additional file 9: Table S6) and used RT-PCR/RACE to amplify the cDNA sequences. As a result, eight Clade A *PP2C* genes were cloned, which were *BnaABI1*, *ABI2*, *HAB1*, *HAB2*, *AHG1, AHG3, HAI2* and *HAI3* according to the nomenclature of their orthologs in Arabidopsis (Table 2). Sequence, domain analysis and phylogenetic analyses of the translated protein sequences revealed that these eight PP2C proteins showed very high identity/similarity to their orthologs in Arabidopsis and also harbored typical domains and motifs (Additional file 10: Figure S4).

### Identification of BnaCPK interacting proteins

**Figure 4:**
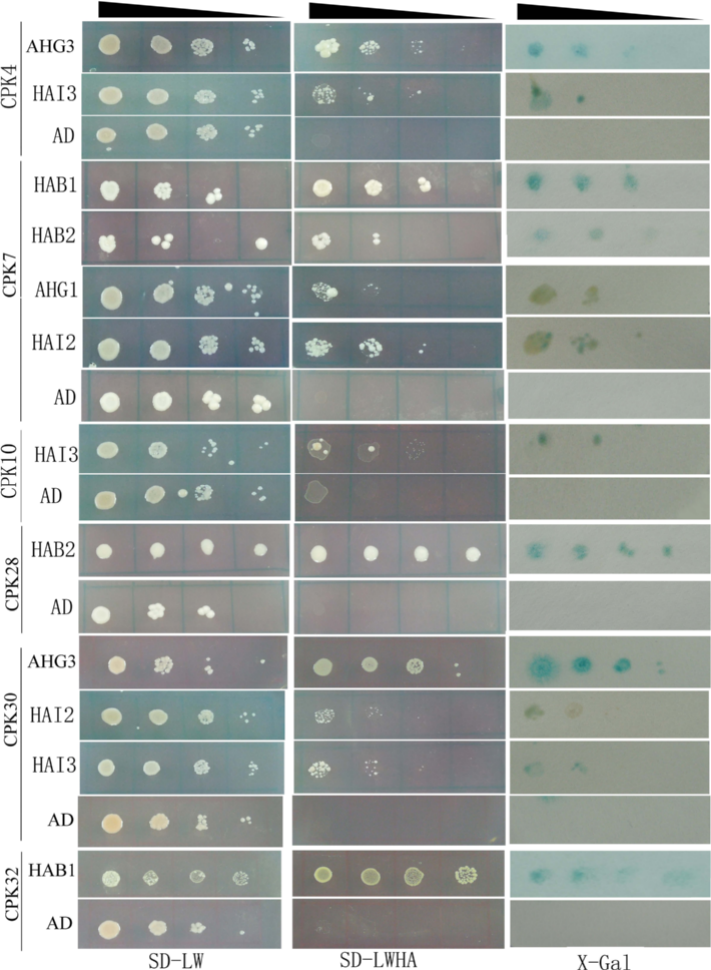
**Yeast two-hybrid analysis of interactions between CPK and PP2C (Clade A) proteins in canola.** The yeast cells of strain AH109 harboring the indicated plasmid combinations were grown on either the nonselective (SD-LW) or selective (SD-LWHA) media, followed by β-galactosidase assay (X-Gal staining). Decreasing cell densities in the dilution series are illustrated by narrowing triangles. Non-interacting BnaCPK-BnaPP2C or BnaCPK-BnaABF/AREB/ABI combinations were not shown here. AD is the empty pGADT7 vector.

To test the interactions between canola CPKs and Clade A bZIPs or PP2Cs, we subcloned the coding regions of canola *CPK*s and *bZIPs* or *PP2Cs* into the GAL4-BD (pGBKT7) and GAL4-AD (pGADT7) vectors, respectively. After transformation into the yeast strain AH109, interactions were detected by growth on several types of media: non-selective (SD-Leucine-Tryptophan, SD-LW) medium; selective medium lacking histidine, leucine, tryptophan supplemented with 5 mM of 3-aminotriazole (3-AT) (SD-LWH+3-AT); or selective media lacking histidine, leucine, tryptophan, adenine hemisulfate (SD-LWHA). We observed that colony growth on the two types of selective media (SD-LWH+3-AT, SD-LWHA) was slightly different from each and thus only those yeast colonies that grew on the most stringent media SD-LWHA were scored as having interacting protein partners. The authenticity and strength of the interactions were further examined by a titration assay and X-gal staining (Figure 4).

As shown in Figure 4, we found that BnaCPK4 interacted with BnaAHG3 and BnaHAI3; BnaCPK7 with BnaHAB1, BnaHAB2, BnaAHG1 and BnaHAI2; BnaCPK10 with BnaHAI3 only; BnaCPK28 with BnaHAB2 only; BnaCPK30 with BnaAHG3, BnaHAI2 and BnaHAI3; BnaCPK32 with BnaHAB1 only, among the eight Clade A PP2C proteins tested. In contrast, we did not observe any yeast colony growth with the empty vector control, i.e. BnaCPKs and pGADT7 combinations. The other BnaCPKs did not show any interaction with any of the eight BnaPP2Cs (data not shown), indicating specificity of interaction between BnaCPKs and BnaPP2Cs. A recent study identified that Arabidopsis CPK11 interacts with AHG1 and AHG3 in Y2H [58]; however, canola CPK11 did not show interaction with BnaAHG1 and BnaAHG3, suggesting that BnaCPK11 function may differ from its ortholog in Arabidopsis, which needs to be studied further.

Surprisingly, we failed to detect any interaction between any of the 23 BnaCPKs with any of the five Clade A bZIP TFs (BnaABF1, BnaABF3, BnaABF4, BnaAREB3, BnaABI5) through streaking or titration assay on both non-selective SD-LW and selective SD-LWHA media within 7 d at 30°C (data not shown). Although yeast colonies harboring all BnaCPK-bZIP combinations grew well on control SD-LW medium plates within 2-3 days, no growth was detected on selective SD-LWHA media within 7 d (data not shown). Interestingly, purported substrates for AtCPK4 and AtCPK11, ABF1 and ABF4 [32], were not identified as putative substrates in Y2H screens [59]. A recent report demonstrated that use of the constitutively active versions of the CPK baits improved the recovery of positive interacting proteins relative to the wild-type kinases [59]. Although this may raise a question on the correlation between Y2H and kinase-substrate, we hypothesized that expression levels of plant TFs in the heterogeneous yeast may not be high enough to assure interactions, a prerequisite of every Y2H system. However, this would be an issue worth carefully examining in detail.

### Bimolecular fluorescence complementation (BiFC) analysis of interactions

To assay the interactions between canola CPKs and Clade A bZIP TFs or PP2Cs *in planta*, we employed YFP (yellow fluorescence protein)-based BiFC. For this purpose, we fused the coding regions of *BnaCPK4, -7, -10, -28* and *BnaABF1, ABF3, ABF4, AREB3, ABI5A, HAB1, HAB2* to the 5′ and 3′ of YFP half gene, respectively. After agrobacteria-mediated infiltration into *N. benthamiana* leaf epidermal cells, we examined the presence of yellow fluorescence signals 4 d later in at least three independent leaf discs.

**Figure 5:**
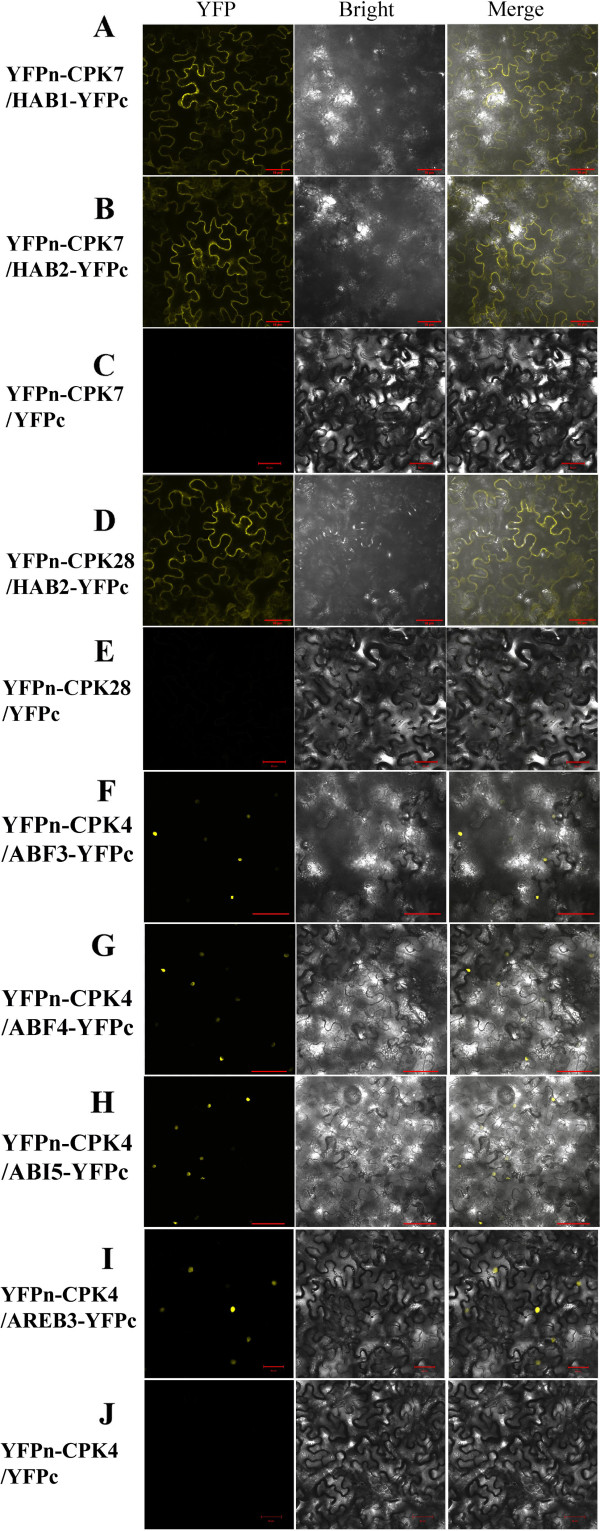
**Bimolecular fluorescence complementation (BiFC) analysis of interactions between BnaCPKs and Clade A BnaPP2Cs or BnabZIP TFs co-expressed in *N. benthamiana *leaf cells.** The coding regions of *BnaCPKs* and *BnaPP2Cs* or *BnabZIPs* were fused to the N- and C-terminal halves of YFP, respectively. The plasmid combinations are indicated at the left side. **(A)** YFP_N_-CPK7 coexpressed with HAB1-YFPc; **(B)** YFP_N_-CPK7 with HAB2-YFPc; **(C)** YFP_N_-CPK7 with YFPc; **(D)** YFP_N_-CPK28 with HAB2-YFPc; **(E)** YFP_N_-CPK28 with YFPc; **(F)** YFP_N_-CPK4 with ABF3-YFPc; **(G)** YFP_N_-CPK4 with ABF4-YFPc; **(H)** YFP_N_-CPK4 with ABI5-YFPc; **(I)** YFP_N_-CPK4 with AREB3-YFPc; **(J)** YFP_N_-CPK4 with YFPc. The fluorescence of YFP was observed by confocal laser microscopy. The left panel is YFP fluorescence, the middle bright field and the right represents an overlay of the two images. Bar = 50 μm.

The results showed that in leaf cells co-expressing BnaCPK7 and BnaHAB1 or HAB2, evident YFP signal appeared in leaf cells (Figure 5A, B), while in the control test, in which the PP2C partner was missing, no yellow signal was observed (Figure 5C). YFP signals also appeared when BnaCPK28 and BnaHAB2 were co-infiltrated (Figure 5D), but no signal of reconstructed YFP appeared in the control cells (Figure 5E). These data suggest our above results of screening of BnaCPK-PP2C interactions are reliable.

We then used BiFC to examine the interactions between BnaCPKs and BnaABF/AREB/ABI5 TFs *in planta*. The results showed that YFP signal appeared in infiltrated *N. benthamiana* cells when BnaCPK4 and BnaABF3, -4, ABI5 or AREB3 were co-infiltrated (Figure 5F-I). However, co-expression of BnaCPK4 and BnaABF1 proteins did not yield any YFP signal, indicating specificity of interaction between BnaCPK4 and TFs. In Arabidopsis, ABI5 is a core player in ABA signaling and acts downstream of ABI3 (maize homologue VP1) and ABI5 is regulated by both ubiquitin-mediated degradation and sumoylation [60-62]. Our identification of AREB3 and ABI5 as novel interaction partners of CPK4 is novel and would be a good starting point for further study of their regulation in ABA and drought signaling. Since AtCPK10 was found to interact with ABF4 in Y2H assay [30], we also wanted to know whether this interaction was conserved and applicable to canola counterparts. However, the BiFC assay showed that BnaCPK10 and BnaABF1, -3, -4, AREB3 or ABI5 did not interact at all, as no yellow signal was observed (data not shown), suggesting that AtCPK10 and BnaCPK10 may play different roles at least in ABA signaling. Taken together, the results from BiFC assay confirmed part of the interacting protein partners between BnaCPKs and BnaPP2Cs on one hand; on the other hand, BiFC also discovered some novel interacting partners, which were not identified in Y2H. We are currently investigating the CPK4-AREB3 and CPK4-ABI5 signaling pathway in both canola and Arabidopsis using a reverse genetic strategy. We think testing the interactions between more BnaCPKs and ABF/AREB/ABI5 TFs in *N. benthamiana* should help us to better understand the signaling pathways and possible mechanisms of interactions. Considering the limitation of Y2H in detection interactions between BnaCPKs and TFs, we are utilizing BiFC to test the interactions between CPK3, -5, -6, -11 etc and ABF/AREB/ABI5 TFs *in planta* one-by-one, so as to decipher the signaling pathways involved.

## Conclusions

The calcium ion plays a central role as second messenger in eukaryotic signal transduction. CPKs are chimeras containing a Ca^2+^-sensor domain and a protein kinase effector domain within the same molecule. Although functions of a few members of Arabidopsis or rice *CPKs* have been studied, many remain uncharacterized to date [15,21]. In addition, the inner mechanisms and the direct targets of most CPKs are still not very clear. So far, no systematic analysis of the CPK family genes has been reported in the important oil crop canola.

In the present study, we carried out a detailed survey of the CPK gene family in canola and analyzed them on the bases of phylogenetic relationship, conserved protein motif, gene duplication, interaction partners and expression profiles to abiotic stress and the stress hormone ABA treatments. A total of 25 CPK genes were identified from canola, with cDNA sequences of 23 CPK genes cloned. We found that 20 out of 21 canola *CPKs* assayed exhibited differential responses to multiple stress treatments, suggesting that they were major convergence points for cross talk between different signal transduction pathways. On the other hand, multiple CPKs seemed to be necessary to coordinate responses to each specific stress stimulus. For instance, transcription of eight, seven, seven, seven canola CPK genes appeared to be affected by salt, cold, heat and drought treatments, respectively, indicating the involvement of multiple CPK signaling pathways in response to abiotic stress. In addition, we analyzed the protein partners of BnaCPKs using Y2H and identified novel interacting proteins, including PP2Cs. We also confirmed part of the interactions between canola CPKs and PP2Cs through BiFC and, tested and identified novel ABA-relevant TFs (ABF3 and ABI5) as CPK4 substrates. Therefore, our genomic, bioinformatic and experimental analyses of the CPK family genes and proteins provide a solid foundation for the further functional characterization of the CPK network involved in decoding calcium signals under different stress conditions. The elucidation of the precise roles of BnaCPK genes, however, requires the use of other experimental approaches including overexpression and/or RNAi silencing. Taken together, by further understanding the functions and underlying mechanisms of the CPK network in canola through virus-induced gene silencing (VIGS) and overexpression, as underway in our lab, we would be able to elucidate how the canola CPK network enables integration of multiple signals of the plant’s environment and coordinates downstream responses to stresses such as toxic ion exposure, extreme temperatures and nutrient deprivation.

## Methods

### Identification of CPK, bZIP and PP2C genes in canola (*Brassica napus* L.)

Before our work, only one putative calcium-dependent kinase 6 mRNA (partial cds) was deposited in GenBank (accession number DQ071551.1). For the identification of expressed sequence tags (ESTs) coding CDPK homologues in canola, a method reported previously was essentially followed [63]. In brief, the full-length cDNA of 34 Arabidopsis CPK genes downloaded from TAIR10 (http://www.arabidopsis.org) were used to search against the EST database at NCBI(National Centre for Biotechnology Information; http://www.ncbi.nlm.nih.gov/dbEST/index.html) and also the DFCI oilseed_rape Gene Index (http://compbio.dfci.harvard.edu/tgi/cgi-bin/tgi/gimain.pl?gudb=oilseed_rape). Only these EST hits with e-value lower than 1e-4 were retrieved. These ESTs were further cleaned, clustered, and assembled. The resultant contigs and singletons were reciprocally searched against Arabidopsis database to identify the best hit among all the 34 AtCPK genes, for each contig and singleton, which is the putative ortholog. Similarly, the cDNA sequences of Clade A bZIP TFs and PP2C genes were obtained.

### Plant material, growth condition and cDNA cloning

Wild type canola (DH12075) plants were grown in a soil mix in the greenhouse with a photoperiod of 16 h light (PHILIPS fluorescent tubes with a light intensity of approximately 90 μE m^-2^ s^-1^)/8 h dark, and a temperature of 22°C day and night for 10 days. Young leaves were harvested for RNA isolation using the Plant RNA kit (Omega bio-tek, USA). RNA integrity was checked by electrophoresis on an agarose gel and quantified using the NanoDrop 1000 (Thermo Scientific, USA). cDNAs were obtained from 2.5 μg of total RNA by using H-MMLV reverse transcriptase and oligo(dT)_18_ (Fermentas, USA). PCR including RACE was conducted as described previously [63]. In short, amplification was performed in a 50 μL final volume including 0.5 μL of cDNA template, 1 × PrimeSTAR buffer, 200 μM deoxynucleotide triphosphates (dNTPs) (TaKaRa, Japan), 400 nM of forward and reverse primers, and 1 unit of PrimeSTAR HS DNA polymerase (TaKaRa). The primers are synthesized in Sangon (Sangon, Shanghai, China) and are listed in Additional file 11: Table S7. The PCR conditions included an initial denaturation at 94°C for 3 min, followed by 35 cycles of 94°C for 30 s, 50°C for 15 s, 72°C for 1 min per kb, with a final extension at 72°C for 8-10 min. PCR products were gel purified using the BioSpin Gel Extraction Kit (Bioer, China) and cloned into pJET1.2 vector supplied with the CloneJET PCR cloning kit (Fermentas) and sequenced from the two ends using BigDye reagent on an ABI3730 sequencer (Applied Biosystems, USA). Sequencing results were analyzed in DNASTAR (DNASTAR Inc., USA) and the identity of CDPK was confirmed through a PROSITE scan (http://prosite.expasy.org/).

### Phylogenetic tree construction and bioinformatics

The CPK gene sequences of various species were obtained from the NCBI or identified from Phytozome V9.0 (http://www.phytozome.com) database through an HMM-based search. The species encompass major land plant lineages including the bryophyte *Physcomitrella patens*[64], the lycophyte *Selaginella moellendorffii* (Sm), and several mono- and eudicotyledonous angiosperms, for example, the eudicot *Arabidopsis thaliana* (At), and the monocots *Oryza sativa* (Os), *Sorghum bicolor* (Sb) and *Brachypodium distachyon* (Bd) as well as a single celled green alga, *Chlamydomonas reinhardtii* (Cr) (Additional file 4: Table S3). To trace the origins of CPK gene families, we also performed a HMM-based search for any possible CPK genes from a marine green alga *Ostreococcus tauri* (*Ot*), which is the smallest free-living eukaryote [65] and also from a pico-eukaryotic (bacterial-sized) prasinophytic green alga *Ostreococcus lucimarinus*, which has one of the highest gene densities known in eukaryotes [66]. As a result, we identified six and four CPK genes from these two organisms, respectively (Additional file 4: Table S3). The protein sequences were then predicted from the genes using the DNASTAR software. To differentiate CPK proteins from other related proteins, such as CPK-related proteins (CRKs), we carefully examined the domain of each sequence and excluded CRK from subsequent analyses.

The phylogenetic tree of CPK from various species were constructed as described previously [63]. In brief, the predicted amino acid sequences of CPK were aligned using ClustalX1.83 program. By using the maximum parsimony algorithm implemented in MEGA5.1, the phylogenetic trees were constructed. The numbers of EF-hand Ca^2+^ binding motifs were detected through SMART program (http://smart.embl-heidelberg.de/smart/set_mode.cgi?NORMAL=1). The myristoylation and palmitoylation motifs were predicted by Myristoylator (http://web.expasy.org/myristoylator/) and CSS-Palm 3.0 (http://csspalm.biocuckoo.org/), respectively. The pairwise identity and similarity of sequences were calculated by program MatGAT v2.02 (http://bitincka.com/ledion/matgat/).

### Subcellular localization and confocal microscopy

The coding regions of BnaCPK genes were amplified by high-fidelity *Pfu*-mediated PCR from the cDNA plasmid in pJET1.2 vector with primers listed in Additional file 11: Table S7. Both pCsGFPBT (GenBank: DQ370426) and pYJGFP vectors with a Gly-Ala rich peptide linker between CDSs and sGFP were used in this study. The modified GFP vector, pYJGFP was made by insertion of multiple cloning sites between *Nco* I and the coding region of the sGFP gene and was sequenced to confirm its reading frame. The N-terminal sequence coding the first 15 amino acids of Arabidopsis calcineurin B-like protein 1 (CBL1) was constructed in-frame before the mCherry gene in a binary vector pYJmCherry, which was modified from a transient expression vector, pBS-mCherry. This CBL1n was demonstrated to be localized at the plasma membrane (PM) [48]. After confirmation by sequencing, these constructs and p19 protein of tomato bushy stunt virus were transferred into *Agrobacterium tumefaciens* GV3101 for infiltrating into the leaves of *N. benthamiana*[67]. The leaf discs near the injection site were cut 2 days after infiltration and the lower epidermis was observed with water as the imaging medium. GFP and mCherry were excited at 488 and 561 nm, respectively, under A1R confocal microscope (Nikon, Japan). All figures show representative images from three independent experiments.

### Abiotic stress treatments and quantitative RT-PCR (qRT-PCR)

Wild type double haploid canola (DH12075) plants were grown in the soil mix in a greenhouse with a photoperiod for 16 h light/8 h dark. 18 d old plants were used for various stress treatments except for drought treatment. Salinity stress was induced by irrigating with 200 mM NaCl into the soil. Cold and heat treatments were applied by putting the plants in 4°C and 37°C, respectively. ABA was applied through spraying plants with 50 μM (±)-ABA (Invitrogen, USA) and oxidative stress was performed by spraying with 10 μM Paraquat (methyl viologen, Sigma-Aldrich, USA). Drought was applied to 14 d old plants through withholding water until evident or severe wilting phenotypes appeared. Above-ground tissues were harvested at 6 and 24 h time points after treatments and flash-frozen in liquid nitrogen before used for RNA extraction. For LK treatment, 18-day-old hydroponically cultured canola seedlings were treated with ½ × MS [containing 1.89 mM K^+^] or a modified ½ × MS solution containing only 100 μM K^+^, with roots harvested 6 and 24 h later. The growth and treatments were repeated three times independently during three continuous days.

The quantitative reverse transcriptase PCR (qRT-PCR) was essentially performed as described earlier [67]. Total RNA was isolated from chemically treated and non-treated canola tissues using Plant RNA kit (Omega, USA). After RNA were quantified by NanoDrop1000 and the integrity was checked on 1% (w/v) gel, and was transcribed into cDNA by using RevertAid H minus reverse transcriptase (Fermentas) and Oligo(dT)_18_ primers (Fermentas). Quantitative reverse transcriptase PCR (qRT-PCR) was performed using 10-fold diluted cDNA and *SYBR Premix Ex Taq*™ kit (TaKaRa, Japan) on the CFX96 real-time PCR machine (Bio-Rad, USA). Primers used for qRT-PCR were designed using the PrimerSelect program implemented in the DNASTAR software, targeting 3′UTR of each gene with an amplicon size between 80-250 bp (Additional file 11: Table S7). The specificity of each pair of primers was checked through regular PCR followed by agarose gel electrophoresis, and also by primer test in CFX96 real-time PCR machine (Bio-Rad, USA) followed by melting curve examination. The amplification efficiency (E) of each primer pair was calculated following that described previously [63,68]. Two endogenous genes (*BnaUP1* and *BnaUBC9*) were used to normalize and calculate the ratios (fold changes). Three independent biological replicates were run and the significance was determined with SPSS (*p* ≤ 0.05).

### Yeast two-hybrid analysis

Yeast two-hybrid analysis was performed using the MatchMaker yeast two-hybrid system (Clontech, USA). Firstly, the coding regions of BnaCPK, BnabZIP and BnaPP2C genes were subcloned into pGBKT7 (BD) and pGADT7 (AD) vectors, respectively. The primers were listed in Additional file 11: Table S7. The plasmids were sequentially transformed into yeast strain AH109 through the lithium acetate method following the protocol described in Yeast Protocols Handbook (Clontech). After plated on three sets of media, SD-Leucine-Tryptophan (SD-LW), SD-Leucin-Tryptophan-Histidine containing 5 mM 3′AT (SD-LWH+3-AT), and SD-Adenine-Histidine-Leucine-Tryptophan (SD-LWHA), the yeast colonies were grown at 30°C for 2-7 d before photographed.

The putative positively interacting transformants were cultivated into YEPD media for serial dilution. In brief, the exponentially grown yeast cells were centrifuged at 5000 g at room temperature and adjusted to OD_600_ = 0.5 with sterilized double-distilled water. Later on, it was diluted 1/10, 1/100 and 1/1000. Two microliters of the aforementioned serial diluted yeast cells were spotted onto SD-LW, SD-LWH+3-AT and SD-LWHA media, grown at 30°C for 2 d (SD-LW plates) or 7 d (SD-LWH+3-AT and SD-LWHA plates) before photographed.

The colony-lift filter assay was conducted following the instruction in the Yeast Protocols Handbook (Clontech). The freshly grown colonies on the selection media (SD-LWHA) were transferred into a sterilized 9 cm filter paper and then flash frozen in liquid nitrogen for 10-20 seconds. After thawing completely, the filter paper which was carried colony side up was transferred into presoaked filter paper in 5 ml of staining buffer containing 5-bromo-4-chloro-3-indolyl-β-D-galactopyranoside (X-gal, Amersco, USA) and β-mercaptoethanol (Amersco) in 90 mm petri dish for 8 h at 37°C. After that, the reaction was stopped and filter paper was dried before being photographed.

### Bimolecular fluorescence complementation (BiFC) assay

To generate the BiFC constructs, the coding regions of *BnaCPK4, -7, -10* and -*28* with stop codons were subcloned into 35S-SPYNE(R)173, and the coding regions of *BnaABF1, ABF3, ABF4, AREB3, ABI5, HAB1* and *HAB2* without stop codons were subcloned into 35S-SPYCE(M) vector [69], using the primers listed in Additional file 11: Table S7. For transient expression, the *Agrobacterium tumefaciens* strain GV3101 carrying each combination of constructs was used together with the p19 strain for infiltration of 5-week-old *Nicotiana benthamiana* leaves. For microscopic observation, the lower epidermis cells of leaves cut 4 d after infiltration were examined on the A1R confocal microscope (Nikon, Japan). All figures show representative images from three independent experiments.

### Accession numbers

The cDNA sequences of BnaCPK, BnabZIP and BnaPP2C genes cloned in this study were deposited in GenBank under the accession No. JX122892-JX122917, KC414029-KC414030, KF169735-KF169741, KF365484-KF365487 and KF974743- KF974749.

## Competing interests

The authors have declared that no competing interests exist.

## Authors’ contributions

YQJ and BY designed, supervised, carried out parts of the experiments, analyzed the data and wrote the manuscript. YQJ, YZ, HZ, WZL, MD, XW, FN, BW and BY performed the gene cloning, qRT-PCR and Y2H assay, HZ, YZ, WZL, MD and YQJ performed the GFP and BiFC analysis. BY, HZ, WL and BW prepared the plant materials for qPCR. MKD provided material, analyzed the data and helped in revising the paper. All authors read and approved the manuscript.

## Supplementary Material

Additional file 1: Table S1 List of ESTs for each CPK genes identified from canola (*Brassica napus* L.). Click here for file

Additional file 2: Table S2 The nucleotide sequence and amino acid residue similarity of BnaCPKs compared with each other and with those from Arabidopsis, rice and Chinese cabbage. Click here for file

Additional file 3: Figure S1 Multiple alignment of 23 CPK protein sequences from canola and motif analysis. Click here for file

Additional file 4: Table S3 CPK genes and encoded proteins from various species used for phylogenetic analysis. Click here for file

Additional file 5: Table S4 CPK genes identified from the Chinese cabbage (*Brassica rapa*) and comparison analysis. Click here for file

Additional file 6: Figure S2 Phylogenetic relationships of CPK proteins from representative species. Click here for file

Additional file 7: Table S5 List of ESTs for bZIP TF (Clade A) genes identified from *Brassica napus*. Click here for file

Additional file 8: Figure S3 Multiple alignment, motif analysis and phylogenetic analysis of Clade A bZIP transcription factors in canola. Click here for file

Additional file 9: Table S6 List of ESTs for PP2C (Clade A) genes identified from *Brassica napus*. Click here for file

Additional file 10: Figure S4 Multiple alignment and phylogenetic analysis of Clade A PP2C proteins in canola. Click here for file

Additional file 11: Table S7 Primers used in this study. Click here for file
