# Supplementary material for: Identification, expression and interaction analyses of calcium-dependent protein kinase (CPK) genes in canola (Brassica napus L.)
Source: BMC Genomics. 2014 Mar 19;15:211. doi: 10.1186/1471-2164-15-211 (PMC4000008; doi:10.1186/1471-2164-15-211)
Supplement: Additional file 3: Figure S1 — Multiple alignment of 23 CPK protein sequences from canola and motif analysis. [file 1471-2164-15-211-S3.pdf]

```
BnaCPK10     1 ---------------------MGNCNVCVRPPNPE-------------------------
BnaCPK30     1 ---------------------MGNCIACVN-FDPE-------------------------
BnaCPK13     1 ---------------------MGNCCRSPAAVARE-------------------------
BnaCPK7      1 ---------------------MGNCCGSPSSATIE-------------------------
BnaCPK8      1 ---------------------MGNCCASPGSDTKT-------------------------
BnaCPK32     1 ---------------------MGNCCGTAGSFPQN-------------------------
BnaCPK24     1 ---------------------MGSCVSSPLKGSPF-------------------------
BnaCPK1      1 MGNTCVGP--SRNGFLHSVSAAMWRPRDDADDSVSQTNGDSVSGELR-----SPLPSEVL
BnaCPK2      1 MGNVCVGPNLSGNGFLQTVSAALWKPRIGAEQASSHGNAQQVPKEAAAEVSKEAVPDQVQ
BnaCPK11     1 ------------------------------------------------------------
BnaCPK4      1 ------------------------------------------------------------
BnaCPK12     1 ------------------------------------------------------------
BnaCPK5      1 ---------------------MGNSCRRSSKDKIHQGNNNNTK-----------------
BnaCPK6      1 ---------------------MGNSCRGSFKDKTYEGNNNNLP-----------------
BnaCPK15     1 ---------------------MG-CFSSKHRRTQSDVVNGNVQRSIPTNQSQTHVPRDVT
BnaCPK21     1 ---------------------MG-CLSSKHRQEE------------------------TA
BnaCPK9      1 ---------------------MGNCFAKNHGLMKP-----------------------QQ
BnaCPK29     1 ----------MDTRTKHFFNRMGFCFSKSQTQEIP------------------------I
BnaCPK17     1 ---------------------MGNCCSGRDSEDGH-------------------------
BnaCPK34     1 ---------------------MGNCCG-RDTGNNN-------------------------
BnaCPK3      1 ---------------------MGHRHSKSKTSSSS-------------------------
BnaCPK18     1 ---------------------MGLCFSSPKAT-RHGTN----------------------
BnaCPK28     1 ---------------------MGLCFSAIRVTGTTSSS----------------------
consensus    1                      .. .

BnaCPK10    15 ---------ESKPKKTNQNRKLNPFTSDFIRSPARTRAP---------------------
BnaCPK30    14 ---------ADSKQTKNQTKKRHVRKPNAYHDPDGLRSHGP-------------------
BnaCPK13    15 ---------DVKSNYSGHDHPRKDSTNGKKSAP---------------------------
BnaCPK7     15 ---------SGHGKPKN---KNNPFHSN---EANGSGAG---------------------
BnaCPK8     15 ---------KAS-RPNT---KSNPFYSEAYTTTNRSGTG---------------------
BnaCPK32    15 ---------DDSNKPKKGKKKQNPFSIDYGLHHNGGGGGG--------------------
BnaCPK24    15 ---------GKRPARRRNNSSSTKTSSNPKTDTSSTSTLSRR------------------
BnaCPK1     54 NKPPEQLTMPKPGGT-NIEIEIHPESKLETQEVTKPEETKEETPAKP-------------
BnaCPK2     61 NKPPEQVTMPNPSSIPEAETKPKPEPEEAKQEVVVQVETSTKPEPKPEPTKPESKPETNP
BnaCPK11     1 ------------------------------------------------------------
BnaCPK4      1 ------------------------------------------------------------
BnaCPK12     1 ------------------------------------------------------------
BnaCPK5     23 --PEDSNSKSSDRST-EIIIPQELPKEAHN--KDPALVIP--------------------
BnaCPK6     23 --EENSITITHVSSVHSPTTEQDFPKEDNNNNKSPVLVLP--------------------
BnaCPK15    39 AHSSIPTNQSQTHVSRDATEPQIPTTTQNHHHNPQEQQESKPVNQPI-------------
BnaCPK21    15 AKPSTPITPAQTHVVPEHRKPQTPPTHHQISAPPPSS-----------------------
BnaCPK9     17 NGQTRSVEVDQTHQDPPSYTPQPRSHTPEKRSSETNQPPP--------------------
BnaCPK29    27 SSSSDSTPPHRYQPLPKPTNPQTQTSTFPTNPKPKPAPPP--------------------
BnaCPK17    15 ---TQDK----GLTDSNAAGPTAEPPVAQSKHAPPSPPPA--------------------
BnaCPK34    14 ---GEP---------TYENGVSAEASVKASRHPPASPPPA--------------------
BnaCPK3     15 ---SSSDNVVHHVKPSGERRGSSGSGSGPVKSSSGSGTGG--------------------
BnaCPK18    17 --HSNPNPPPDIPKPQSQGKGREKVCNQNKKKTKNNKIQWR-------------------
BnaCPK28    18 --RRSSSQTNNNNKKNPKSDKTPPPPPENNNDKPSTTTTKR-------------------
consensus   61
```

```
BnaCPK10    45 --------------------KDAVIPTSHQTKITDKYILGRELGRGEFGITYLCTDRESR
BnaCPK30    46 ------------------IRVLPDVPMSHRTQISDKYILGRELGRGEFGITYLCTDRETR
BnaCPK13    39 ---------------------IRVLTDVPKENIEDRYLLDRELGRGEFGVTYLCIERATR
BnaCPK7     39 -------------------FKLSVLKDPTGHDISSQYDLGREVGRGEFGVTYLCTDIQTG
BnaCPK8     41 -------------------FKLSVLKDPTGHDISLMYDLGREVGRGEFGITYLCTDIKTG
BnaCPK32    46 -------------------LKLTVLSDPTGREIEQKYTLGRELGRGEFGVTYLCTDKETG
BnaCPK24    48 ---------------LIFQPPSRVLPEPIGDGIFLKYELGKELGRGEFGVTHECIEISTR
BnaCPK1    100 -----PKKPKHMKRVTSAGLRTESVLQRKTENFKEFYSLGRKLGQGQFGTTFLCVEKSTG
BnaCPK2    121 ETKPDPSKPKHMRRVSSAGLRTESVLQRKTENFKEFYSLGRKLGQGQFGTTFLCLEKGTG
BnaCPK11     1 ------------MEKANPRRPSNTVLPYQTPRLRDHYLLGKKLGQGQFGTTYLCTEKSTS
BnaCPK4      1 ------------MEKPSSRRPSSSVLPYETPRLRDHYLLGKKLGQGQFGTTYLCTEKSSS
BnaCPK12     1 ---------------MANKSRTRWVLPYTTKNVEDDYFLGQILGQGQFGTTFLCTHKKTA
BnaCPK5     58 --------LKEPIMRRNMDNQAYYVLGHKTPNIRDLYNLSRKLGQGQFGTTYLCTELATG
BnaCPK6     61 --------VKEPFMRRNMDNQAYYVLGHKTPNIRDLYTLSRKLGQGQFGTTYLCTEVATG
BnaCPK15    86 -----EEYQVFKPLKPIALLETETILGKPFEQIKRLYTLGHELGRGQFGITYTCKENSTG
BnaCPK21    52 --------------NPTTVRDHDTILGKPFEDIRKFYSLGKELGRGQFGITYRCREISTG
BnaCPK9     57 -------WRMAAPAPSPKAAKSSSILENAYEDVKLFYTLGKELGRGQFGVTYLCTENSTG
BnaCPK29    67 ---------------SSSGSQIGPILNRPMIDLSALYDLHKELGRGQFGVTYRCTDKSNG
BnaCPK17    48 ----------------TKQGPIGPVLGRPMEDVKASYSLGKELGRGQFGVTHLCTQKATG
BnaCPK34    42 ----------------TKQGPIGPVLGRPMEDIKSSYTLGKELGRGQFGVTHLCTQKATG
BnaCPK3     52 ----------------SRSQQNGRILGKPMEDVKGTYDLGRELGRGQFGVTHLVTHKETK
BnaCPK18    56 --------------HVGGTLFGKRIDFGYARDFDNRYTIGKLLGHGQFGFTYAATDNNNE
BnaCPK28    57 --------------RTGSVPCGKRTEFGYAKDFHDQYSIGKLLGHGQFGYTYVAIHKSNG
consensus  121                         ..      ..  * .....*.*.**.*...... ..

BnaCPK10    85 EALACKSISKRKLRTAVDVEDVRREVSIMSTLPDHPNVVKLRATYEDGESVHLVMELCEG
BnaCPK30    88 EALACKSISKRKLRTAVDVEDVRREVTIMSTIPDHPNVVKLKATYEDNENVHLVMELCEG
BnaCPK13    78 DLLACKSISKRKLRTAVDIEDVKREVAIMKHLPKSSSIVTLKEACEDDSAVHLVMELCEG
BnaCPK7     80 DKYACKSISKKKLRTAVDIGDVRREVEIMRHMPKHPNIVSLKDSFEDDDAVHIVMELCEG
BnaCPK8     82 EKYACKSISKKKLRTAVDIEDVRREVEIMKHMPKHPNIVTLKDAFEDDDAVHIVMELCEG
BnaCPK32    87 EVLACKSILKKKLRTAVDIEDVRREAEIMRHMPEHPNLVTLKETYEDEYAVHLVMELCEG
BnaCPK24    93 ERFACKRISKEKLRTEIDVEDVRREVEIMRSLPKHANIVSFKEAFEDKDAVYLVMEICEG
BnaCPK1    155 KEFACKSIAKRKLLTDEDVEDVRREIQIMHHLSGHQNVISIKGAYEDVVAVHLVMECCAG
BnaCPK2    181 NEYACKSISKRKLLTEEDVEDVRREIQIMHHLAGHPNVISIKGAYEDVVAVHLVMELCSG
BnaCPK11    49 ANYACKSIPKRKLVCREDYEDVWREIQIMHHLSEHPNVVRIKGTYEDSVFVHIVMEVCEG
BnaCPK4     49 ANYACKSIPKRKLVCREDYEDVWREIQIMHHLSEHPNVVRIKGTYEDSVFVHIVMEVCEG
BnaCPK12    46 QKLACKSIPKRKLLCQEDYDDVLREIQIMHHLSEFPNVVRIEGAYEDTSSVHIVMELCEG
BnaCPK5    110 VDYACKSISKRKLISKEDVEDVRREIQIMHHLAGHGNIVTIKGAYEDSLYVHIVMELCAG
BnaCPK6    113 VDYACKSISKRKLISKEDVEDVRREIQIMHHLAGHKNIVTIKGAYEDPLYVHIVMEVCAG
BnaCPK15   141 NTYACKSILKRKLTRKQDRDDVKKEIQIMQHLSGQKNIVEIRGAYEDRQSIHLVMELCGG
BnaCPK21    98 NTYACKSILKRKLISKQDKEDVKREIQIMQYLSGQANIVEIKGAYEDRQSVHLVMELCAG
BnaCPK9    110 KKYACKSISKKKLVTKADKDDMRREIQIMQHLSGQPNIVEFKGAYEDEKAVNLVMELCAG
BnaCPK29   112 REYACKSISKRKLIRQKDIEDVRREVMILQHLTGQPNIVEFRGAYEDKDNLHVVMELCSG
BnaCPK17    92 QQFACKTIAKRKLVNKEDIEDVRREVQIMHHMTGQPNIVELKGAYEDKHSVHLVMELCAG
BnaCPK34    86 LQFACKTIAKRKLVNKEDIEDVRREVQIMHHLTGQPNIVELKGAYEDKHSVHLVMELCAG
BnaCPK3     96 KVFACKSIPTRRLVQSDDIEDVRREVQIMHHLSGHRNIVDLKGAYEDRQSVNLIMELCEG
BnaCPK18   102 DRVAVKRIDKAKMTQPIEIEDVKREVKILQALGGHENVVGFHNVFEDKNYVYIVMELCEG
BnaCPK28   103 DRVAVKRLDKSKMVLPVAVEDVKREVQILKALSGHENVVQFYNAFDDDDYVYIVMELCEG
consensus  181   .*.*.. ......  ...*...*..*........... ......*   ....**.*.*
```

Kinase domain

```
BnaCPK10   145 GELFDRIVAR--GHYTERAAAGVARTIAEVVMMC--------------------------
BnaCPK30   148 GELFDRIVAR--GHYTERAAATVARTIAEVVKMC--------------------------
BnaCPK13   138 GELFDRIVAR--GHYTERAAAGVTKTIVEVVQLC--------------------------
BnaCPK7    140 GELFDRIVAR--GHYTERAAAAVMKTIVEVVQIC--------------------------
BnaCPK8    142 GELFDRIVAR--GHYTERAAAAVMKTILEVVQIC--------------------------
BnaCPK32   147 GELFDRIVAR--GHYTERAAAAVTKTIIEVVQVC--------------------------
BnaCPK24   153 GELFDRIVSR--GHYTERAAASVAKTILEVVKVCEKNSNLLCIFCFFPAFIVVFLCLKVC
BnaCPK1    215 GELFDRIIQR--GHYTERKAAELTRTIVGVVEAC--------------------------
BnaCPK2    241 GELFDRIIQR--GHYTERKAAELARTIVGVLETC--------------------------
BnaCPK11   109 GELFDRIVAK--GHFSEREAVKLIKTILAVVEAC--------------------------
BnaCPK4    109 GELFDRIVSK--GHFSEREAAKLIKTILGVVEAC--------------------------
BnaCPK12   106 GELFDRIVKR--GHYSEREAAKLIKTIVGVVEAC--------------------------
BnaCPK5    170 GELFDRIIQR--GHYSERKAAELTKIIVGVVEAC--------------------------
BnaCPK6    173 GELFDRIIQR--GHYTERKAAELTKIVVGVVEAC--------------------------
BnaCPK15   201 GELFDRIIAQ--GHYSERAAAGVIRSVLNVVQIC--------------------------
BnaCPK21   158 GELFDRIIAQ--GHYSERAAAGIIRSIVNVVQIC--------------------------
BnaCPK9    170 GELFDRIIAK--GHYSERAAASVCRQIVNVVKIC--------------------------
BnaCPK29   172 GELFDRIIKK--GSYSEKEAANIFRQIVNFVHVC--------------------------
BnaCPK17   152 GELFDRIIAK--GHYSERAAASLLRTIVQIIHTC--------------------------
BnaCPK34   146 GELFDRIIAK--GHYSERAAASLLRTIVQIIHTC--------------------------
BnaCPK3    156 GELFDRIIAK--GHYTERAAADLCRQMVMVVHSC--------------------------
BnaCPK18   162 GELLDRILSKKDSRYTEKDAAVVVRQMLKVAAEC--------------------------
BnaCPK28   163 GELLDRILSKKDSRYSEKDAAVVVRQMLKVAGEC--------------------------
consensus  241 ***.***...  ....*..*. . .... ..  *

BnaCPK10   177 HVNGVVHRDLKPENFLFANKKENSALKAIDFGLSVFFKPG--------------------
BnaCPK30   180 HMNGVMHRDLKPENFLFANKKENSALKAIDFGLSVLFKPG--------------------
BnaCPK13   170 HKHGVIHRDLKPDNFLFANKKENSPLKAIDFGLSIFFKPG--------------------
BnaCPK7    172 HKQGVMHRDLKPENFLFANKKETSALKAIDFGLSVFFKPG--------------------
BnaCPK8    174 HKNGVMHRDLKPENFLFANKKENSPLKAIDFGLSVFFKPG--------------------
BnaCPK32   179 HKHGVMHRDLKPENFLFANKKETAPLKAIDFGLSVFFKPG--------------------
BnaCPK24   211 HEHGVIHRDLKPENFLFSNETETAQLKAIDFGLSIFFKPA--------------------
BnaCPK1    247 HSLGVMHRDLKPENFLFVSKDEDSLLKTIDFGLSMFFKPGLDSLYIRTGDLHNGVRVISS
BnaCPK2    273 HSLGVMHRDLKPENFLFVSREEDSLLKTIDFGLSMFFKP---------------------
BnaCPK11   141 HSLGVMHRDLKPENFLFDSPKEDAKLKATDFGLSVFYKPG--------------------
BnaCPK4    141 HSLGVMHRDLKPENFLLDSPNDDAKLKATDFGLSVFYKPG--------------------
BnaCPK12   138 HSLGVMHRDLKPENFLFASCDEDASLKSTDFGLSVFCKPG--------------------
BnaCPK5    202 HSLGVMHRDLKPENFLLVNKDDDFSLKAIDFGLSIFFKPG--------------------
BnaCPK6    205 HSLGVMHRDLKPENFLLVNKDDDFSLKAIDFGLSVFFKPG--------------------
BnaCPK15   233 HFMGVMHRDLKPENFLLSSKDEDAMLKATDFGLSVFIEEG--------------------
BnaCPK21   190 HFMGVVHRDLKPENFLLSSKEENAMLKATDFGLSVFIEEG--------------------
BnaCPK9    202 HFMGVLHRDLKPENFLLSSKDDKALIKATDFGLSVFIEEG--------------------
BnaCPK29   204 HFMGVVHRDLKPENFLLVSADDDSPIKATDFGLSVFIEEG--------------------
BnaCPK17   184 HSMGVIHRDLKPENFLLLSKDENSPLKATDFGLSVFYKPG--------------------
BnaCPK34   178 HSMGVIHRDLKPENFLLLNKDENSPLKATDFGLSVFYKPG--------------------
BnaCPK3    188 HSMGVMHRDLKPENFLFLSKDESSPLKATDFGLSVFFKPG--------------------
BnaCPK18   196 HLRGLVHRDMKPENFLFKSTEENSSLKATDFGLSDFIKPG--------------------
BnaCPK28   197 HLHGLVHRDMKPENFLFKSAKLDSPLKATDFGLSDFIKPG--------------------
consensus  301 * .*..***.**.***. .... . .*..*****......
```

```
BnaCPK10   217 ------------------EKFKEIVGSPYYMAPEVLKRDYGPEVDVWSAGVIIYILLCGV
BnaCPK30   220 ------------------ERFTEIVGSPYYMAPEVLKRDYGPEVDVWSAGVILYILLCGV
BnaCPK13   210 ------------------EKFSEIVGSPYYMAPEVLKRSYGPEIDIWSAGVILYILLCGV
BnaCPK7    212 ------------------EQFNEIVGSPYYMAPEVLRRNYGPEIDVWSAGVILYILLCGV
BnaCPK8    214 ------------------EGFNEIVGSPYYMAPEVLRRHYGPEVDIWSAGVILYILLCGV
BnaCPK32   219 ------------------ERFNEIVGSPYYMAPEVLKRNYGPEVDIWSAGVILYILLCGV
BnaCPK24   251 ------------------QRFNEIVGSPYYMAPEVLRRNYGPEIDVWSAGVILYILLCGV
BnaCPK1    307 ANSFFEIFLIFDPSSLADDVFTDVVGSPYYVAPEVLRKQYGPEADVWSAGVIVYILLSGV
BnaCPK2    312 -----------------DEVFTDVVGSPYYVAPEVLRKRYGPESDVWSAGVIIYILLSGV
BnaCPK11   181 ------------------QYLYDVVGSPYYVAPEVLKKCYGPEIDVWSAGVILYILLSGV
BnaCPK4    181 ------------------QYLYDVVGSPYYVAPEVLKKCYGPEIDVWSAGVILYILLSGV
BnaCPK12   178 ------------------ATFSELVGSAYYVAPEVLHKHYSRECDVWSAGVILYILLCGF
BnaCPK5    242 ------------------QIFTDVVGSPYYVAPEVLLKRYGPEADVWTAGVILYILLSGV
BnaCPK6    245 ------------------QIFKDVVGSPYYVAPEVLLKHYGPEADVWTAGVILYILLSGV
BnaCPK15   273 ------------------KVYRDIVGSAYYVAPEVLRRSYGKEIDIWSAGIILYILLCGV
BnaCPK21   230 ------------------KVYRDIVGSAYYVAPEVLRRSYGKEIDIWSAGVILYILLSGV
BnaCPK9    242 ------------------KVYRDIVGSAYYVAPEVLRRRYGKEVDIWSAGIILYILLSGV
BnaCPK29   244 ------------------KVYKDVVGSAYYVAPEVLYRNYGKEIDVWSAGVMLYILLCGV
BnaCPK17   224 ------------------EVFKDIVGSAYYIAPEVLKRKYGPEADIWSIGVMLYILLCGV
BnaCPK34   218 ------------------EEFKDIVGSAYYIAPEVLKRKYGPEADIWSIGVMLYILLSGV
BnaCPK3    228 ------------------DKFKDLVGSAYYVAPEVLKRNYGPEADIWSAGVILYILLSGV
BnaCPK18   236 ------------------MKFQDIVGSAYYVAPEVLKRRSGPESDVWSIGVITYILLCGR
BnaCPK28   237 ------------------KKFHDIVGSAYYVAPEVLKRRSGPESDVWSIGVITYILLCGR
consensus  361                     . ..***.**.*****.. ...*.*.*..*...****.*.

BnaCPK10   259 PPFWAETEQGVALAILRGVIDFKRDPWPQISESAKSLVRQMLNPDPTKRLTAQQVLAHPW
BnaCPK30   262 PPFWAETEQGVALAILRGVLDFKRDPWSQISESAKSLVKQMLNPDPTKRLTAQQVLDHPW
BnaCPK13   252 PPFWAESEQGVAQAILRGIIDFKREPWPNISETAKSLVRQMLEPDPKRRLTAKQVLEHPW
BnaCPK7    254 PPFWAETEQGVAQAIIRSVIDFKRDPWPRVSESAKDLVRKMLEPDPKKRLSAAEVLEHTW
BnaCPK8    256 PPFWAETEQGVAQAIIRSVIDFKRDPWPRVSDTAKDLVRRMLEPDPKKRLSAAEVLEHPW
BnaCPK32   261 PPFWAETEQGVAQAIIRSQLDFRRDPWPKVSEHAKDLIRKMLDPDQKRRLTAQQVLDHPW
BnaCPK24   293 PPFWAETEEGIAHAIVRGNIDFERDPWPKVSREAKELVKNMLDANPYSRLTVQEVLEHPW
BnaCPK1    367 PPFWAESEQGIFEQVLHGDLDFSSDPWPSISESAKDLVRKMLVRDPKKRLTAHQVLCQPW
BnaCPK2    355 PPFWAETEQGIFEQVLHGDLDFSSDPWPSISDGAKDLVQKMLVRDPKRRLTAHQVLCHPW
BnaCPK11   223 PPFWAETESGIFRQILQGKLDFKSDPWPTISEAAKDLIYKMLERSPKKRISAHEALCHPW
BnaCPK4    223 PPFWAETESGIFRQILQGKLDFKSDPWPTISEGAKDLIYKMLDRSPKKRISAHEALCHPW
BnaCPK12   220 PPFWDESEFGIFRKILQGKIDFETSPWPSISESAKDLIVKMLEKDPKKRLTAHQVLCHPW
BnaCPK5    284 PPFWAETQQGIFDAVLKGYIDFDSDPWPVISDSAKDLIRRMLCSKPAERLTAHEVLRHPW
BnaCPK6    287 PPFWAETQQGIFDAVLKGDIDFESDPWPVISDSAKDLIRKMLCSNPSERLTAHEVMRHPW
BnaCPK15   315 PPFWAETEKGIFEEIIKGEIDFESQPWPSISESAKDLVRKLLTMDPRKRISAAQALEHPW
BnaCPK21   272 PPFWAENEKGIFDEVVKGEIDFESQPWPSISESAKDLVRKMLTKDPRRRITAAQVLEHPW
BnaCPK9    284 PPFWAETEKGIFDAILEGHIDFESQPWPSISNSAKDLVRKMLTADPKRRISAADVLEHPW
BnaCPK29   286 PPFWGETEKTIFEAVLEGNLDLESSPWPTISESAKDLIRKMLARDPKKRITAAEALAHTW
BnaCPK17   266 PPFWAESENGIFNAILKSHVDFSSDPCPSISPQAKDLVKKMLNSDPKQRLTAAQVLNHPW
BnaCPK34   260 PPFWAESENGIFNAILSGQIDFSTD---------------------------------PW
BnaCPK3    270 PPFWGENETGIFDAILEGKLDFSADPWPDVSNGAKDLVKKMLTYDPKDRLTASEVLNHPW
BnaCPK18   278 RPFWDKTQNGIFNEVMRKKPDFETTPWPTISDGAKDFVKKLLVKEPRARLTAAQALSHSW
BnaCPK28   279 RPFWDRTEDGIFKEVLRNKPDFRRKPWSTISDSAKDFIKKLLVKDPRARLTASQALSHAW
consensus  421 .***........ ..... .*. ...................  ........ ... ..*
```

```
BnaCPK10   319 VQNAKKAPNVPLGDIVRSRLKQFSMMNRFKKKVLRVIAEHLSIQEVEVIKDMFSLMDEDN
BnaCPK30   322 IQNAKKAPNVPLGDIVRSRLKQFSMMNRLKKKALRVIAEHLSIQEVEVIRDMFTLMDDDN
BnaCPK13   312 IQNAKKAPNVPLGDVVKSRLKQFSVMNRFKRKALRVIAEFLSSQEVEDIKEMFNKMDTDK
BnaCPK7    314 ILNAKKAPNVSLGETVKARLKQFSVMNKLKKRALRVIAEHLSVEEAAGIKEAFEMMDVNK
BnaCPK8    316 IQNAKKAPNVSLGETVKARLKQFSVMNKLKKRALRVIAEHLSVEEVAGIKEAFEMMDSKK
BnaCPK32   321 LQNANTAPNVSLGETVRARLKQFTVMNKLKKRALRVIAEHLSDEEASGIREGFRIMDTSQ
BnaCPK24   353 IQNAERAPNVNLGDGVRTKIQQFLLMNRFKKKVLRVVADNLPNEEIESIIQMFQTMDTDK
BnaCPK1    427 VQVDGVAPDKPLDSAVLSRMKQFSAMNKFKKMALRVIAESLSEEEIAGLKEMFNMIDADK
BnaCPK2    415 VQIDGVAPDKPLDSAVLSRMKQFSAMNKFKKMALRVIAESLSEEEIAGLKEMFKMIDADN
BnaCPK11   283 IVDEEAAPDKPLDPAVLSRLKQFSQMNKIKKMALRVIAERLSEEEIGGLKELFKMIDTDN
BnaCPK4    283 IVDEQAAPDKPLDPAVLSRLKQFSQMNKIKKMALRVIAERLSEEEIGGLKELFKMIDTDN
BnaCPK12   280 IVDDKVAPDKPLDCAVLSRLKNFSAMNKLKKMAFRVIAERLSEEEIGGLKELFRMIDTDN
BnaCPK5    344 ICENGVAPDRALDPAVLSRLKQFSAMNKLKKMALKVIAESLSEEEIAGLREMFQAMDTDN
BnaCPK6    347 ICENGVAPDRALDPAVLSRLKQFSAMNKLKKMALKVIAESLSEEEIAGLRAMFEAMDTDN
BnaCPK15   375 IR-GGEAPDNPIDSAVLSRMKQFRAMNKLKKLALKVIAESLSEEEIKGLKTMFANMDTDQ
BnaCPK21   332 IK-GGEAPDKPIDSAVLSRMKQFRAMNKLKKLALKVIAESLSEEEIKGLKTMFANMDTDK
BnaCPK9    344 LREGGEASDKPIDSAVLSRMKQFRAMNKLKKLALKVIAENINTEEIQGLKAMFANIDTDN
BnaCPK29   346 LT-DSEVSDKPIDSAVLIRMKQFRAMNKLKKLALKVIAENLSEEEIKGLKHMFKNIDTDG
BnaCPK17   326 IKEDGEAPDVPLDNAVMSRLKQFKAMNNFKKVALRVIAGCLSEEEIMGLKEMFKGMDTDS
BnaCPK34   287 IKEDGEAPDVPLDNAVMSRLKQFKAMNNFKKVALRVIAGCLSEEEIMGLKEMFKGMDTDD
BnaCPK3    330 IKEDGEASDKPLDNAVLSRMKQFRAMNKLKKMALKVIAENLSEEEIIGLKEMFKALDTDK
BnaCPK18   338 VREGGEASEIPIDISVLENMRQFVKFSRLKQIALKALATTIDEDELDDLRDQFDAIDIDK
BnaCPK28   339 VREGGNATDIPVDISVLNNLRQFVRYSRLKQFALRALASTLDEAEISDLRDQFDAIDVDK
consensus  481 .  .. ... ... .*......*......*.......*. ....*. .....*  .*..
                                                             -----------
                                                             EF-hand motif 1

BnaCPK10   379 DGRITYLELKAGLQKVG-SQLGEPEIKMLMEVADVDGNGFLDYGEFVAVIIHLQKIENDE
BnaCPK30   382 DGKITYPELRAGLKKVG-SQLGEPEIKMLMEVADVNGNGCLDYGEFVAVIIHLQKMENDE
BnaCPK13   372 DGIVTIEELKAGLRDFG-TQLAESEVQMLIEAVDTKGKGTLDYGEFVAVSLHLQKVANDE
BnaCPK7    374 RGKINLEELKYGLQKAG-QQIADADLQILMEATDVDGDGTLNYGEFVAVSVHLKKMANDE
BnaCPK8    376 TGKINLEELKHGLHKLGQQQIPDTDLQILMEAADVDGDGTLNYGEFVAVSVHLKKMANDE
BnaCPK32   381 RGKINIDELKIGLQKLG-HNIPQDDIQILMDAGDTDKDGYLDCDEFIAISVHLRKMGNDE
BnaCPK24   413 NGHLTFEELRDGLKKIG-QVCPDGDVKMLMDAADTDGNGTLSCEEFVTLAIHLKRMGCDE
BnaCPK1    487 SGQITFEELKAGLKRVG-ANLKESEILDLMQAADVDNSGTIDYKEFIAATLHLNKIERED
BnaCPK2    475 SGQITFEELKAGLKRVG-ANLKESEILDLMQAADVDNSGTIDYKEFIAATLHLNKIEKED
BnaCPK11   343 SGTITFEELKAGLKRVG-SELMESEIKSLMDAADIDNSGTIDYGEFLAATLHMNKMEREE
BnaCPK4    343 SGTITFEELKAGLKRVG-SELMESEIKSLKDAADIDNSGTIDYGEFLAATLHMNKMEREE
BnaCPK12   340 SGTITFEELKDTVKRVG-ADLMESEIQELLRSADVDENGSIDYGEFLAATIHLNKLEREE
BnaCPK5    404 SGAITFDELKAGLRKYG-STLKDTEIHDLMEAADVDNSGTIDYSEFIAATIHLNKLEREE
BnaCPK6    407 SGAITFDELKAGLRRYG-STLKDTEIQDLMEAADVDNSGTIDYSEFIAATIHLNKLDREE
BnaCPK15   434 SGTITYEELKTGLARLG-SKLSEAEVKQLMEAADVDGNGTIDYIEFISATMHRYRLDRDE
BnaCPK21   391 SGTITYEELKTGLTRLG-SKLSETEVKQLMEAADVDGNGTIDYFEFISATMHRYKLDRDE
BnaCPK9    404 SGTITYEELKEGLAKLG-SKLTEAEVKQLMDAADVDGNGSIDYIEFITATMHRHRLESDE
BnaCPK29   405 SGTITFDELRTGLHRLG-SKLTESEIKQLMEAADVDKSGTIDYIEFITATMHRHRLEKEE
BnaCPK17   386 SGTITLEELRQGLAKQG-TRLSEYEVQQLMEAADADGNGTIDYGEFIAATMHINRLDREE
BnaCPK34   347 SGTITLEELRQGLAKQG-TRLSEYEVQQLMEAADADGNGTIDYGEFIAATMHINRLDREE
BnaCPK3    390 NGIVTLEELRTGLPKLG-SKISEAEIKQLMEAADMDGDGSIDYLEFISATMHMNRIERED
BnaCPK18   398 NGSISLEEMRQALAKDLPWKLKDARVAEILQAIDSNTDGLVDFTEFVVATLHVNQLEEHD
BnaCPK28   399 NGVISLEEMRQALAKDLPWKLKESRVAEILEAIDSNTDGLVDFTEFVAAALHVHQLEEHD
consensus  541 .* ....*.. ...... . . ....  .....*... *.... **.....*........
                 ------------          -----------------------
                 EF-hand motif 1       EF-hand motif 2
```

```
BnaCPK10   438 ------LFKLAFMFFDKDGSTYIELDELREALTDELGEPD--VSVLNDIMREVDSDKDGR
BnaCPK30   441 ------HFRQAFMFFDKDGSGYIESDELRRALTDELGEPD--NSVLIDIMREVDTDKDGR
BnaCPK13   431 ------HLRKAFSYFDKDGNGYILPEELCEALKEDGGDDC--VDVANDIFQEVDTDKDGR
BnaCPK7    433 ------HLHKAFNFFDQNQSGYIETEELREALNDELDETSS-EEVIAAIMQDVDTDKDGR
BnaCPK8    436 ------HLHKAFSFFDKNQSDYIEIEELREALNDEVDTSS--EEVIAAIMQDVDTDKDGR
BnaCPK32   440 ------HLKKAFAFFDQDNNGYIEIEELREALSDEVGTSE---EVVDAIIRDVDTDKDGR
BnaCPK24   472 ------HLQQAFKYFDKNGNGSIELDELKEALFDDDKLGHGGDQWIKDIFFDVDLNKDGR
BnaCPK1    546 ------HLFAAFTYFDKDGSGYITPDELQQACEEFGVED----VRIEEMMSDVDQDNDGR
BnaCPK2    534 ------HLFAAFSYFDKDGSGFITPDELQHACEEFGVED----ARIEEMMRDVDQDKDGR
BnaCPK11   402 ------NLVAAFSYFDKDGSGYITIDELQSACTEFGLCD----TPLDDMIKEIDLDNDGK
BnaCPK4    402 ------NLVVAFSYFDKDGSGYITIDELQLACTEFGLCD----TPLDDMIKEIDLDNDGR
BnaCPK12   399 ------NLVAAFSFFDKDGSGYITVDELQQALKEFGIND----SHLDEMIKDIDQDNDGQ
BnaCPK5    463 ------HLVAAFQYFDKDGSGYITIDELQQACVEHSMTD----VFLEDIIKEVDQNNDGK
BnaCPK6    466 ------HLVSAFQYFDKDGSGYITIDELQQSCVEHGMTD----VFLEDVIKEVDQDNDGR
BnaCPK15   493 ------HLFKAFQYFDKDNSGFITMDELKSAMKEYGMGDE---TSIKEVIAEVDTDNDGR
BnaCPK21   450 ------HVYKAFQHFDKDNSGHITRDELESAMKEYGMGDE---ASIKEVISEVDTDNDGR
BnaCPK9    463 ------NLYKAFQHFDKDGSGYITIDELEVALKEYGMGDD---ATIKEVLSDVDSDNDGR
BnaCPK29   464 ------HLLEAFKYFDKDRSGYITRDELKHSMTQYGMGDD---ATIDEVINDVDTDNDGR
BnaCPK17   445 ------HLYSAFQHFDKDNSGYITMEELEQALREFGMSDG---RDIKEIISEVDGDNDGR
BnaCPK34   406 ------HLYSAFQHFDKDNSGYITMEELEQALREFGMNDG---RDIKEIISEVDGDNDGR
BnaCPK3    449 ------HLYTAFQYFDKDNSGYITMEELEQAMKKYNMGDD---KSIKEIIAEVDTDRDGK
BnaCPK18   458 SDKWEQRSRAAFEKFDVDRDGFITPEELRLQTGLKGSIEP--------LLEEADIDEDGR
BnaCPK28   459 SEKWQLRSRAAFEKFDIDKDGYITPEELRLHTGLRGSIDP--------LLDEADIDRDGK
consensus  601       ..  ** .**......*...**  .. .... .      . ... ..* ..**.
```

EF-hand motif 3 EF-hand motif 4

```
BnaCPK10   490 INYDEFVTMMKAGTDWRKASRQYSRERFKSLSINLMKDGSLHLHDALTGQSVPV------
BnaCPK30   493 INYDEFVVMMKAGTDWRKASRQYSRERFKSLSINLMKDGSLHLHDALTGQSVAV------
BnaCPK13   483 ISYEEFAAMMKTGTDWRKASRHYSRGRFNSLSIKLMKDGSLNLGNE--------------
BnaCPK7    486 ISYEEFAAMMKAGTDWRKASRQYSRERFNSLSLKLMRDGSLQLAGEA-------------
BnaCPK8    488 ISYEEFAAMMRAGTDWRKASRQYSRERFNSLSLKLMRDGSLQLEGET-------------
BnaCPK32   491 ISYEEFVTMMKTGTDWRKAFRQYSRERFNSISLKLMQDASLHANGDTR------------
BnaCPK24   526 ISFDEFRAMMKSGTDWKMASRQYSRALLNALSIKMFKEDVGDNGPKSYSMEFPLARKKAK
BnaCPK1    596 IDYNEFVAMMQKGSIAGGGGPVKMGG-------LEKSFSNIALKL---------------
BnaCPK2    584 IDYNEFVAMMQKGSIMGG--PMKMG--------LEKSIS-ISLKH---------------
BnaCPK11   452 IDFSEFTAMMKKGDGVG--RSRTMMKNLNFNIADAFGVDEQSAQKSDD------------
BnaCPK4    452 IDFSEFTAMMKKGDGVG--RSRTMMKNLNFNIADAFGVEETSTAETDDKPN---------
BnaCPK12   449 IDYGEFVAMMRKGNGSGGIGRRTMRNTLNFANWKVASSSQMARNLS--------------
BnaCPK5    513 IDYGEFVEMMQKGNAGVG--RRTMRN--------SLNISMRDA-----------------
BnaCPK6    516 IDYGEFVAMMQKGNAGIG--RRTMRN--------SLNISMRDA-----------------
BnaCPK15   544 INYEEFCAMMRSGTTQPQQGKTSSIPL---------------------------------
BnaCPK21   501 INFEEFCAMMRSGTTQPQ-GKLFPFH----------------------------------
BnaCPK9    514 INYEEFCAMMRSGNPQQQQQQQPRLF----------------------------------
BnaCPK29   515 INYEEFVAMMTKGTTDHSDAKLIR------------------------------------
BnaCPK17   496 INYDEFVAMMRKGNPDQIPKKRRELSFK--------------------------------
BnaCPK34   457 INYEEFVAMMRKGNPDPNPKKRREMSFK--------------------------------
BnaCPK3    500 INYEEFVAMMKKGHPELVTNRRRVNM----------------------------------
BnaCPK18   510 ISIHEFRRLLRSASLKPRTVKSPPGYQLSRKM----------------------------
BnaCPK28   511 ISLHEFRRLLRTASISSPRVPSTAGHRIPR------------------------------
consensus  661 * ..**..... ..      .
```

EF-hand motif 4

```
BnaCPK10       -------------------------
BnaCPK30       -------------------------
BnaCPK13       -------------------------
BnaCPK7        -------------------------
BnaCPK8        -------------------------
BnaCPK32       -------------------------
BnaCPK24   586 LLDAPKNKSMELVHSKTYKPSGLRY
BnaCPK1        -------------------------
BnaCPK2        -------------------------
BnaCPK11       -------------------------
BnaCPK4        -------------------------
BnaCPK12       -------------------------
BnaCPK5        -------------------------
BnaCPK6        -------------------------
BnaCPK15       -------------------------
BnaCPK21       -------------------------
BnaCPK9        -------------------------
BnaCPK29       -------------------------
BnaCPK17       -------------------------
BnaCPK34       -------------------------
BnaCPK3        -------------------------
BnaCPK18       -------------------------
BnaCPK28       -------------------------
consensus  721
```

Figure S1. Multiple alignment of 23 CDPK protein sequences from canola and motif analysis. Hyphens indicate gaps introduced to maximize the sequence alignment. Identical residues are highlighted in black, and similar residues are highlighted in gray. The Ser/Thr kinase domain and four EF-hand motifs are shown by a thick line and dashed line under the sequences, respectively. The multiple alignment was performed using the ClustalX1.83 and illustrated by BOXSHADE (http://www.ch.embnet.org/software/BOX_form.html).
