# Supplementary material for: Identification, expression and interaction analyses of calcium-dependent protein kinase (CPK) genes in canola (Brassica napus L.)
Source: BMC Genomics. 2014 Mar 19;15:211. doi: 10.1186/1471-2164-15-211 (PMC4000008; doi:10.1186/1471-2164-15-211)
Supplement: Additional file 6: Figure S2 — Phylogenetic relationships of CPK proteins from representative species. [file 1471-2164-15-211-S6.pdf]

(A)

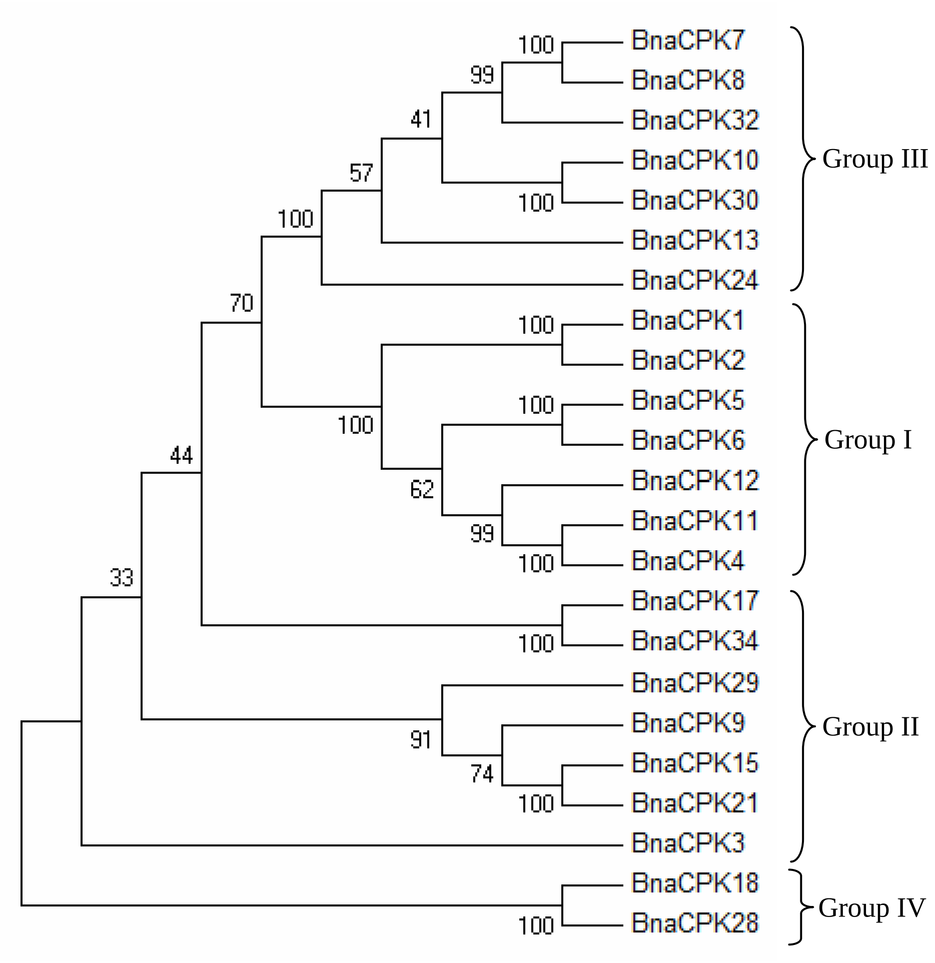
BnaCPK7
BnaCPK8
BnaCPK32
BnaCPK10
BnaCPK30
BnaCPK13
BnaCPK24
BnaCPK1
BnaCPK2
BnaCPK5
BnaCPK6
BnaCPK12
BnaCPK11
BnaCPK4
BnaCPK17
BnaCPK34
BnaCPK29
BnaCPK9
BnaCPK15
BnaCPK21
BnaCPK3
BnaCPK18
BnaCPK28
Group III
Group I
Group II
Group IV
100
99
41
57
100
70
100
100
100
62
99
100
44
33
100
91
74
100
100

(B)

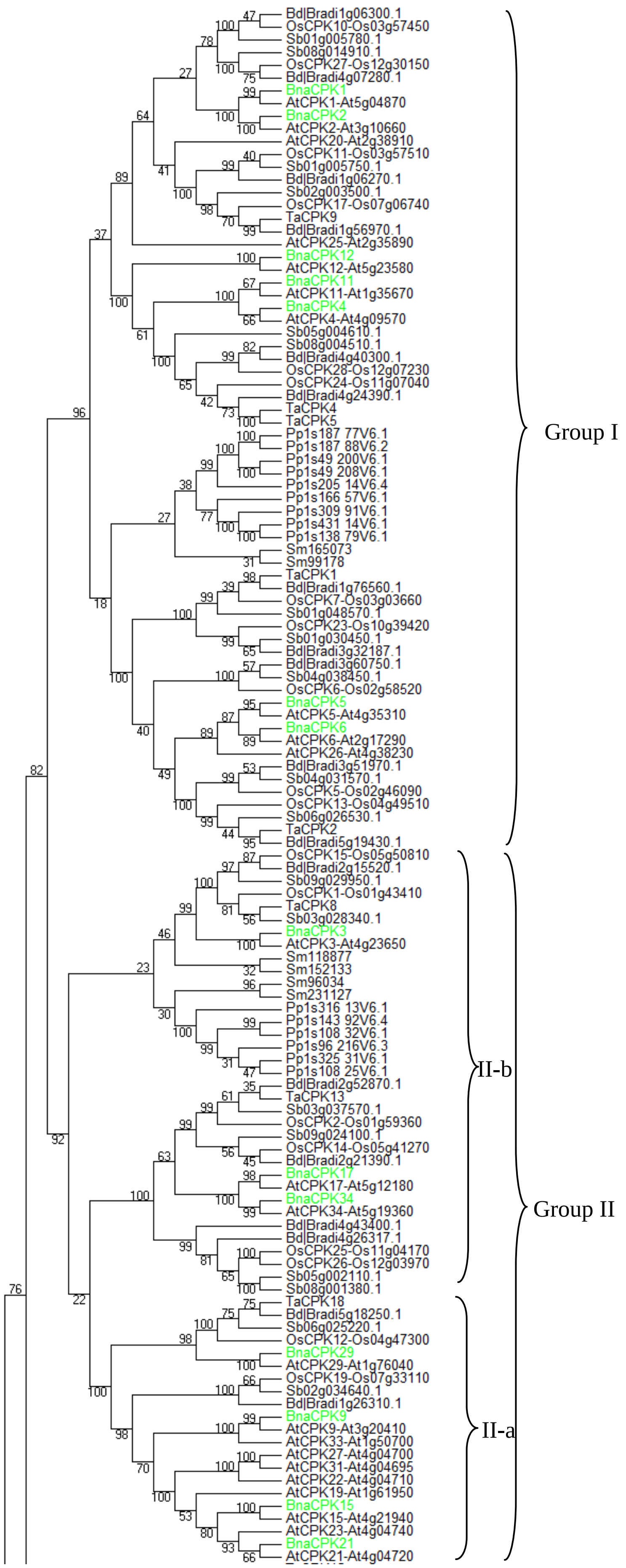
Bd|Bradi1g06300.1
OsCPK10-Os03g57450
Sb01g005780.1
Sb08g014910.1
OsCPK27-Os12g30150
Bd|Bradi4g07280.1
BnaCPK1
AtCPK1-At5g04870
BnaCPK2
AtCPK2-At3g10660
AtCPK20-At2g38910
OsCPK11-Os03g57510
Sb01g005750.1
Bd|Bradi1g06270.1
Sb02g003500.1
OsCPK17-Os07g06740
TaCPK9
Bd|Bradi1g56970.1
AtCPK25-At2g35890
BnaCPK12
AtCPK12-At5g23580
BnaCPK11
AtCPK11-At1g35670
BnaCPK4
AtCPK4-At4g09570
Sb05g004610.1
Sb08g004510.1
Bd|Bradi4g40300.1
OsCPK28-Os12g07230
OsCPK24-Os11g07040
Bd|Bradi4g24390.1
TaCPK4
TaCPK5
Pp1s187 77V6.1
Pp1s187 88V6.2
Pp1s49 200V6.1
Pp1s49 208V6.1
Pp1s205 14V6.4
Pp1s166 57V6.1
Pp1s309 91V6.1
Pp1s431 14V6.1
Pp1s138 79V6.1
Sm165073
Sm99178
TaCPK1
Bd|Bradi1g76560.1
OsCPK7-Os03g03660
Sb01g048570.1
OsCPK23-Os10g39420
Sb01g030450.1
Bd|Bradi3g32187.1
Bd|Bradi3g60750.1
Sb04g038450.1
OsCPK6-Os02g58520
BnaCPK5
AtCPK5-At4g35310
BnaCPK6
AtCPK6-At2g17290
AtCPK26-At4g38230
Bd|Bradi3g51970.1
Sb04g031570.1
OsCPK5-Os02g46090
OsCPK13-Os04g49510
Sb06g026530.1
TaCPK2
Bd|Bradi5g19430.1
OsCPK15-Os05g50810
Bd|Bradi2g15520.1
Sb09g029950.1
OsCPK1-Os01g43410
TaCPK8
Sb03g028340.1
BnaCPK3
AtCPK3-At4g23650
Sm118877
Sm152133
Sm96034
Sm231127
Pp1s316 13V6.1
Pp1s143 92V6.4
Pp1s108 32V6.1
Pp1s96 216V6.3
Pp1s325 31V6.1
Pp1s108 25V6.1
Bd|Bradi2g52870.1
TaCPK13
Sb03g037570.1
OsCPK2-Os01g59360
Sb09g024100.1
OsCPK14-Os05g41270
Bd|Bradi2g21390.1
BnaCPK17
AtCPK17-At5g12180
BnaCPK34
AtCPK34-At5g19360
Bd|Bradi4g43400.1
Bd|Bradi4g26317.1
OsCPK25-Os11g04170
OsCPK26-Os12g03970
Sb05g002110.1
Sb08g001380.1
TaCPK18
Bd|Bradi5g18250.1
Sb06g025220.1
OsCPK12-Os04g47300
BnaCPK29
AtCPK29-At1g76040
OsCPK19-Os07g33110
Sb02g034640.1
Bd|Bradi1g26310.1
BnaCPK9
AtCPK9-At3g20410
AtCPK33-At1g50700
AtCPK27-At4g04700
AtCPK31-At4g04695
AtCPK22-At4g04710
AtCPK19-At1g61950
BnaCPK15
AtCPK15-At4g21940
AtCPK23-At4g04740
BnaCPK21
AtCPK21-At4g04720
Group I
II-b
Group II
II-a

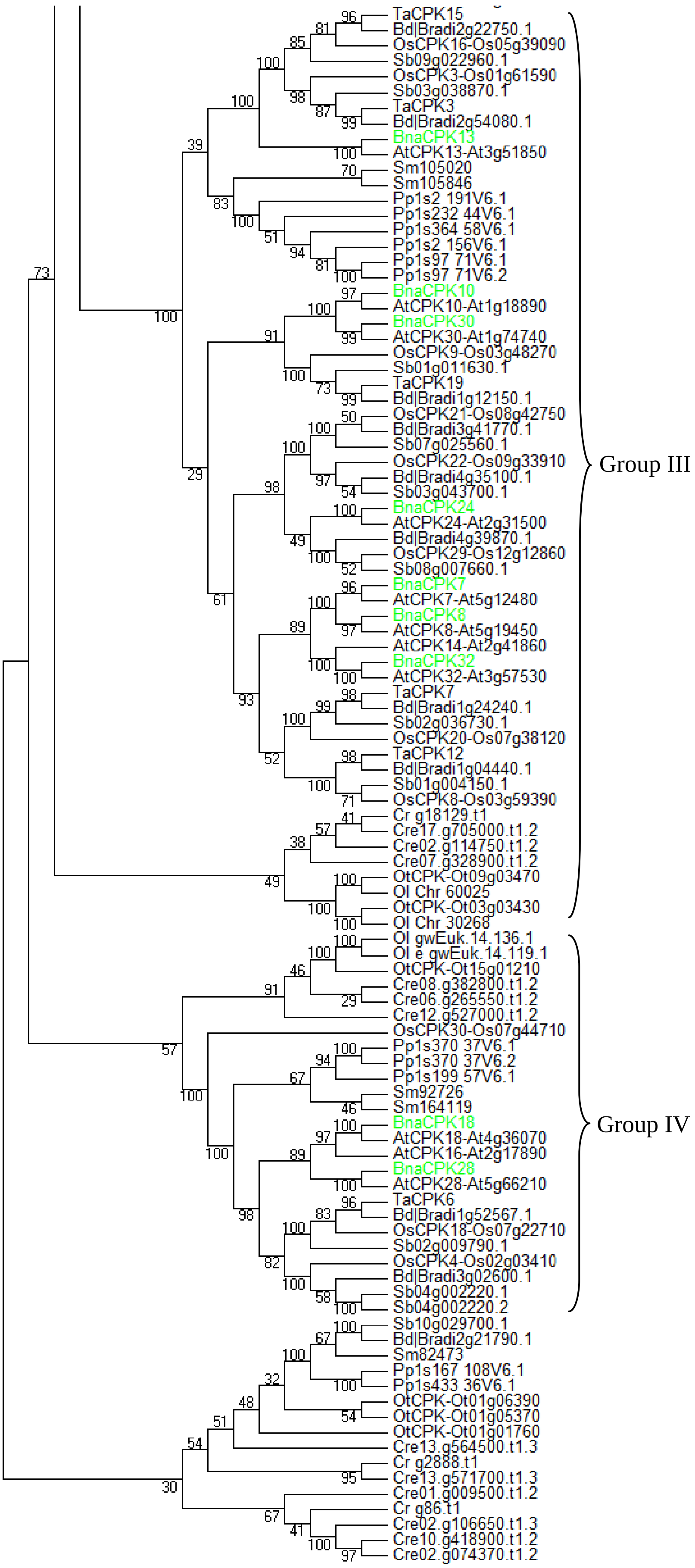
TaCPK15
Bd|Bradi2g22750.1
OsCPK16-Os05g39090
Sb09g022960.1
OsCPK3-Os01g61590
Sb03g038870.1
TaCPK3
Bd|Bradi2g54080.1
BnaCPK13
AtCPK13-At3g51850
Sm105020
Sm105846
Pp1s2 191V6.1
Pp1s232 44V6.1
Pp1s364 58V6.1
Pp1s2 156V6.1
Pp1s97 71V6.1
Pp1s97 71V6.2
BnaCPK10
AtCPK10-At1g18890
BnaCPK30
AtCPK30-At1g74740
OsCPK9-Os03g48270
Sb01g011630.1
TaCPK19
Bd|Bradi1g12150.1
OsCPK21-Os08g42750
Bd|Bradi3g41770.1
Sb07g025560.1
OsCPK22-Os09g33910
Bd|Bradi4g35100.1
Sb03g043700.1
BnaCPK24
AtCPK24-At2g31500
Bd|Bradi4g39870.1
OsCPK29-Os12g12860
Sb08g007660.1
BnaCPK7
AtCPK7-At5g12480
BnaCPK8
AtCPK8-At5g19450
AtCPK14-At2g41860
BnaCPK32
AtCPK32-At3g57530
TaCPK7
Bd|Bradi1g24240.1
Sb02g036730.1
OsCPK20-Os07g38120
TaCPK12
Bd|Bradi1g04440.1
Sb01g004150.1
OsCPK8-Os03g59390
Cr g18129.t1
Cre17.g705000.t1.2
Cre02.g114750.t1.2
Cre07.g328900.t1.2
OtCPK-Ot09g03470
Ol Chr 60025
OtCPK-Ot03g03430
Ol Chr 30268
Group III
Ol gwEuk.14.136.1
Ol e gwEuk.14.119.1
OtCPK-Ot15g01210
Cre08.g382800.t1.2
Cre06.g265550.t1.2
Cre12.g527000.t1.2
OsCPK30-Os07g44710
Pp1s370 37V6.1
Pp1s370 37V6.2
Pp1s199 57V6.1
Sm92726
Sm164119
BnaCPK18
AtCPK18-At4g36070
AtCPK16-At2g17890
BnaCPK28
AtCPK28-At5g66210
TaCPK6
Bd|Bradi1g52567.1
OsCPK18-Os07g22710
Sb02g009790.1
OsCPK4-Os02g03410
Bd|Bradi3g02600.1
Sb04g002220.1
Sb04g002220.2
Group IV
Sb10g029700.1
Bd|Bradi2g21790.1
Sm82473
Pp1s167 108V6.1
Pp1s433 36V6.1
OtCPK-Ot01g06390
OtCPK-Ot01g05370
OtCPK-Ot01g01760
Cre13.g564500.t1.3
Cr g2888.t1
Cre13.g571700.t1.3
Cre01.g009500.t1.2
Cr g86.t1
Cre02.g106650.t1.3
Cre10.g418900.t1.2
Cre02.g074370.t1.2

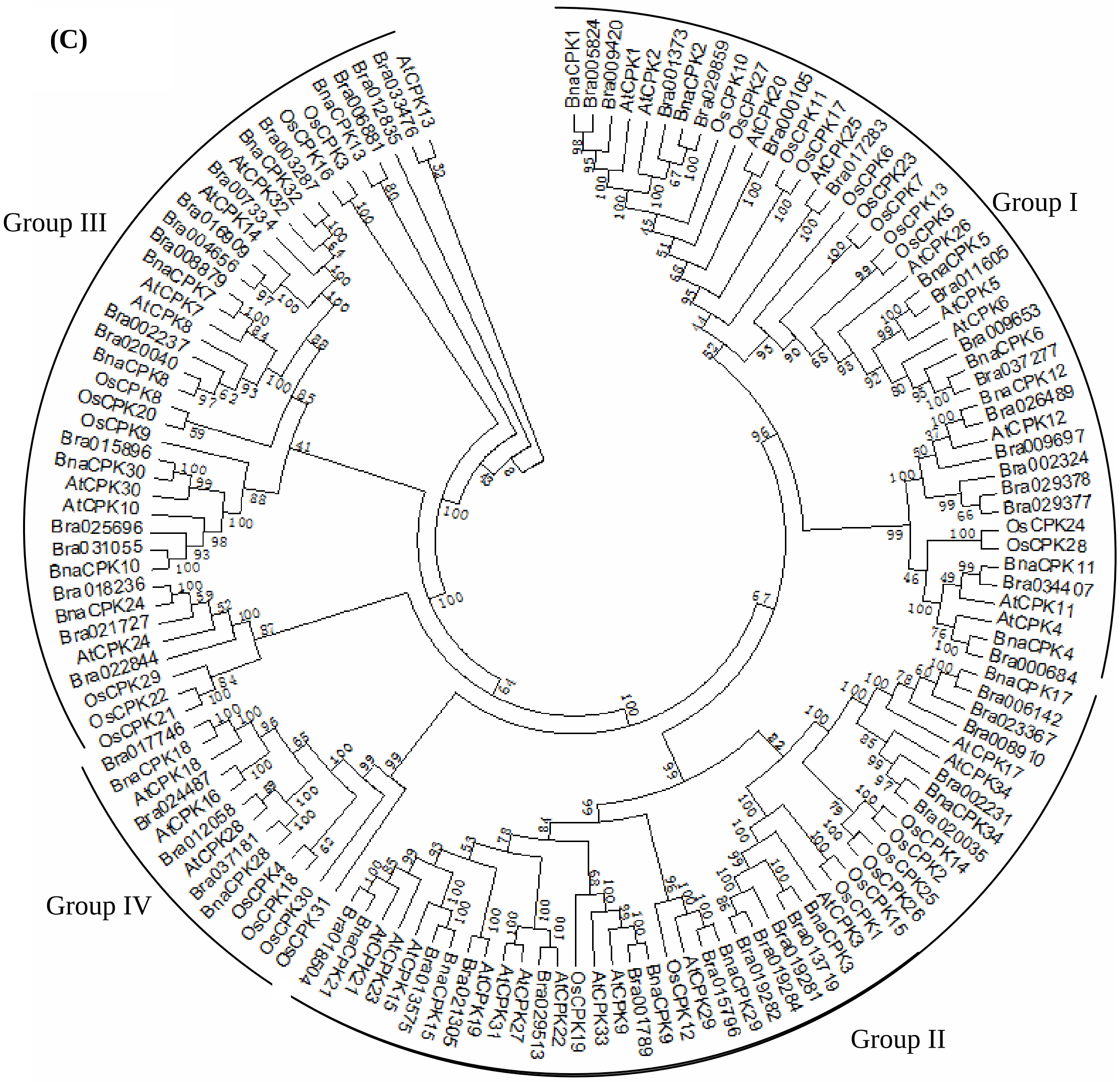

Figure S2. Phylogenetic relationships of CPK proteins from representative species.

(A) Phylogenetic relationships among canola CPKs. (B) Phylogenetic relationships of CPK proteins from representative species**.** (C) Phylogenetic relationships of CPK proteins from Arabidopsis, rice, canola and Chinese cabbage. Protein sequences were aligned using ClustalX (v1.83) and a maximum parsimony (MP) bootstrap consensus tree was drawn using MEGA5.1. The CPKs can be clustered into four major groups (I-IV). The percentage of replicate trees is shown on the branches and it is calculated in the bootstrap test (1000 replicates) for the associated taxa being clustered together. The 23 BnaCPKs reported in this study are highlighted in green. Each taxon was named by a two to three letters representing the species followed by locus ID. At, *Arabidopsis thaliana*; Bd, *Brachypodium distachyon;* Bna, *Brassica napus;* Bra,

Brassica rapa; Cr, *Chlamydomonas reinhardtii*; Os, *Oryza sativa*; Pp, *Physcomitrella patens*； Sb, *Sorghum bicolor*; Sm, *Selaginella moellendorffii ;* Ta, *Triticum aestivum*; Ol, *Ostreococcus lucimarinus*; Ot, *Ostreococcus tauri.*
