# Supplementary material for: Identification, expression and interaction analyses of calcium-dependent protein kinase (CPK) genes in canola (Brassica napus L.)
Source: BMC Genomics. 2014 Mar 19;15:211. doi: 10.1186/1471-2164-15-211 (PMC4000008; doi:10.1186/1471-2164-15-211)
Supplement: Additional file 8: Figure S3 — Multiple alignment, motif analysis and phylogenetic analysis of Clade A bZIP transcription factors in canola. [file 1471-2164-15-211-S8.pdf]

(A)

```
AtABF1       1 -------MGTHIDINNLGG-----DTS-RGN-----ESKPLARQSSLYSLTFDELQSTLG
BnaABF1      1 -------MGTHINFNSLGV-----DSS-GGNGSDNNQSKPLGRQSSLYSLTFDELQSTLG
BnaABF3      1 -------MGSRMNFVDVVR-----DEVINAKQPALGSGLPLTRQNSVFSLTFDEFQNSWG
BnaABF4      1 -------MGTHINFNNLGG-----GGGNQMKPTDTTN--PLARQSSLYSLTFDELQSTLL
BnaAREB3     1 -------MDSQREPRS---------------------HQSLNRQGSLYSLTLDEVQTHLG
BnaABI5      1 MVSRETEMMSEREVETSTAQARQNGGAGGGGGGENHPFSSLGRQSSIYSLTLDEFQHALC
consensus    1        *...........     .    .    .    .*.**.*..***.**.*....

AtABF1      43 E-PGKDFGSMNMDELLKNIWTAEDT-QAFMTTTSSV------------------------
BnaABF1     48 E-PGKEFGSMNMDELLKNIWTAEETTQPIMTTTSSI------------------------
BnaABF3     49 GGIGKDFGSMNMDELLKNIWTAEESHSMMANNTVMN------------------------
BnaABF4     47 GGPGKDFGSMNMDELLKSIWTAEEAHAMTMNPSSTA------------------------
BnaAREB3    33 S-SGKALGSMNLDELLKSVCSVETN-----------------------------------
BnaABI5     61 E-NGKNFGSMNMDEFLVSIWNAEENNNNSHQAAAASHPVPPNHNGFNNNNNGGTESGVFG
consensus   61 . .**..****.**.*. ....*.. .  .. ...

AtABF1      77 ----AAPG--PSGFVPGGNGLQ-RQGSLTLPRTLSQKTVDEVWKYLNSKEGSNGNT----
BnaABF1     83 ----ASVQQPSSGFAPGGGGLVQRQGSLTLPRTLSQKTVDEVWKHLMSKDSCN-------
BnaABF3     85 ----SGGGLSVGVGGEVGGCGLQRQGSIALPRTISQKRVDDVWKELMKEEDDTG------
BnaABF4     83 ----TAAAQPG------GGIPLQRQGSLTLPITITQKTVDEVWKCLFTKDGNMVGSI---
BnaAREB3    57 -----------QPPSMAVNEGLSRQGSLTLPRDLSKKTVEEVWKDIQQ------------
BnaABI5    120 GGGSSGNQGVNKKPGIAKQPSLPRQGSLTLPAPLCRKTVEEVWSEIHRGGGSGDGDNSNG
consensus  121     .. .      . ...  ..****..**.....*.*..**... ....

AtABF1     126 ---------------GTDALERQQTLGEMTLEDFLLRAGVVKEDNTQQ----NENSSSGF
BnaABF1    132 ---------------GRDAPERQETLGEMTLEDFLLRAGVVKEDVNCS----QQNQN---
BnaABF3    135 ---------------ASGVPQRQQTLGEMTLEEFLLKAGVVREEPQQHVERLDNFNGGFY
BnaABF4    130 -----------GGGSESNAPHRQQTLGEITLEEFLLRAGVVRED--------NNNNNGFY
BnaAREB3    94 --------DKNGGGSGHERRDKQPTLGEMTLEDLLLKAGVVTET----------------
BnaABI5    180 RSTSSSNGQNNANNGGGESAARQPTFGEMTLEDFLVKAGVVREHPTNP------------
consensus  181            .   .......*.*.**.***..*..****.*.  .     ....   .

AtABF1     167 YANNGAAGLEFGFGQPNQNS--ISFNGNNSSMIMNQAPGLGLKVG------GTMQQQQQP
BnaABF1    170 --NCVSTGVGIGFGQPNQNN--IWFKGNNSSMILNQA--------------------QQP
BnaABF3    180 G-FGSNAGLGSAPNEFGPNQPYGVTVRPDLLKIQAQPLQMQQRQQLIPKQVEPFPKQTTV
BnaABF4    171 SNNGAPGGLGFGFGQPNQNN-ISFNNGTNDSMILKQPPHQQFQQQSSQPQLQPRQQLNQP
BnaAREB3   130 -----------VPGSNHGN----------------------------------------G
BnaABI5    228 KPMLNPTPTSVIPASTQQQQLYGVFSGGGDPSFPVGVGDYGKRTG--------------G
consensus  241   .   .... . .......    . . . .... .      .              ...

AtABF1     219 HQQQLQQPHQRLPPTIFPKQANVTFAAPVNMVNRGLFETSADGPANSNMGGAGGTVTATS
BnaABF1    206 HQQQLQQPHQRLPPTIYPKHANVTFSAPV-------------------MGKAGVSVASTS
BnaABF3    239 AFSNTVGLDNRSQPPTQYQEVKPSILGVR--------PMNNNNLQAVDFKTG-VTVEAVS
BnaABF4    230 HPPQQQHPHQRMPQTIFPKQANVAFAGAGNN------AGNNNNNGLGSFGGGGVTVAATS
BnaAREB3   139 SAGMEQNITQVAPWVQYHQLPSMPQPQSFM----------------------PYPVADMQ
BnaABI5    274 GGGGYQQAPPVQPGVCYGGGGGFGAGGQQMG-----------------MVGPLSPVSSDG
consensus  301 .  . ............. .......                     .......*....

AtABF1     279 PG-------TSSAENN-TWSSPVPYVFG-RGRRSNTG-LEKVVERRQKRMIKNRESAARS
BnaABF1    247 PGG------TNSAENNAAWSSPVPYVFGGQGRRSNTGVVEKVVERRQKRMIKNRESAARS
BnaABF3    290 PGSQMSPDLTPKSNMDASLSPPVPYMFG-RARKTGAV-LEKVIERRQKRMIKNRESAARS
BnaABF4    284 PG---------TSSAENNSLSPVPYVLN-RGRRSNTG-LEKVIERRQRRMIKNRESAARS
BnaAREB3   177 AMVS-------QSSLMGGLSDTQTP---GRKRVASGEVVEKTVERKQKRMIKNRESAARS
BnaABI5    317 LGHG-------QVDNIGGQYGVDMGGLRGRKRVVDGP-VEKVVERRQRRMIKNRESAARS
consensus  361 ..       . .... .. ............*..... .**..**.*.************
```

basic region

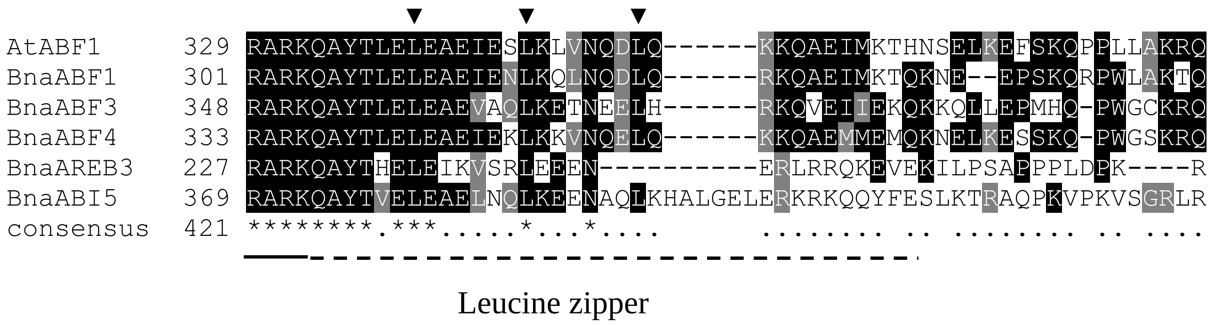
AtABF1 329 RARKQAYTLELEAEIESLKLVNQDLQ------KKQAEIMKTHNSELKEFSKQPPLLAKRQ
BnaABF1 301 RARKQAYTLELEAEIENLKQLNQDLQ------RKQAEIMKTQKNE--EPSKQRPWLAKTQ
BnaABF3 348 RARKQAYTLELEAEVAQLKETNEELH------RKQVEIIEKQKKQLLEPMHQ-PWGCKRQ
BnaABF4 333 RARKQAYTLELEAEIEKLKKVNQELQ------KKQAEMMEMQKNELKESSKQ-PWGSKRQ
BnaAREB3 227 RARKQAYTHELEIKVSRLEEEN----------ERLRRQKEVEKILPSAPPPLDPK----R
BnaABI5 369 RARKQAYTVELEAELNQLKEENAQLKHALGELERKRKQQYFESLKTRAQPKVPKVSGRLR
consensus 421 ********.***.....*...*.... ....
Leucine zipper
AtABF1 383 CLRRTLTGPW
BnaABF1 353 CLRRTLTGPW
BnaABF3 401 CLRRTSTGPW
BnaABF4 386 CLRRTLTGPW
BnaAREB3 273 QLRRTSSAPF
BnaABI5 429 TLMRNPSCPL
consensus 481 .*.*....*.

(B)

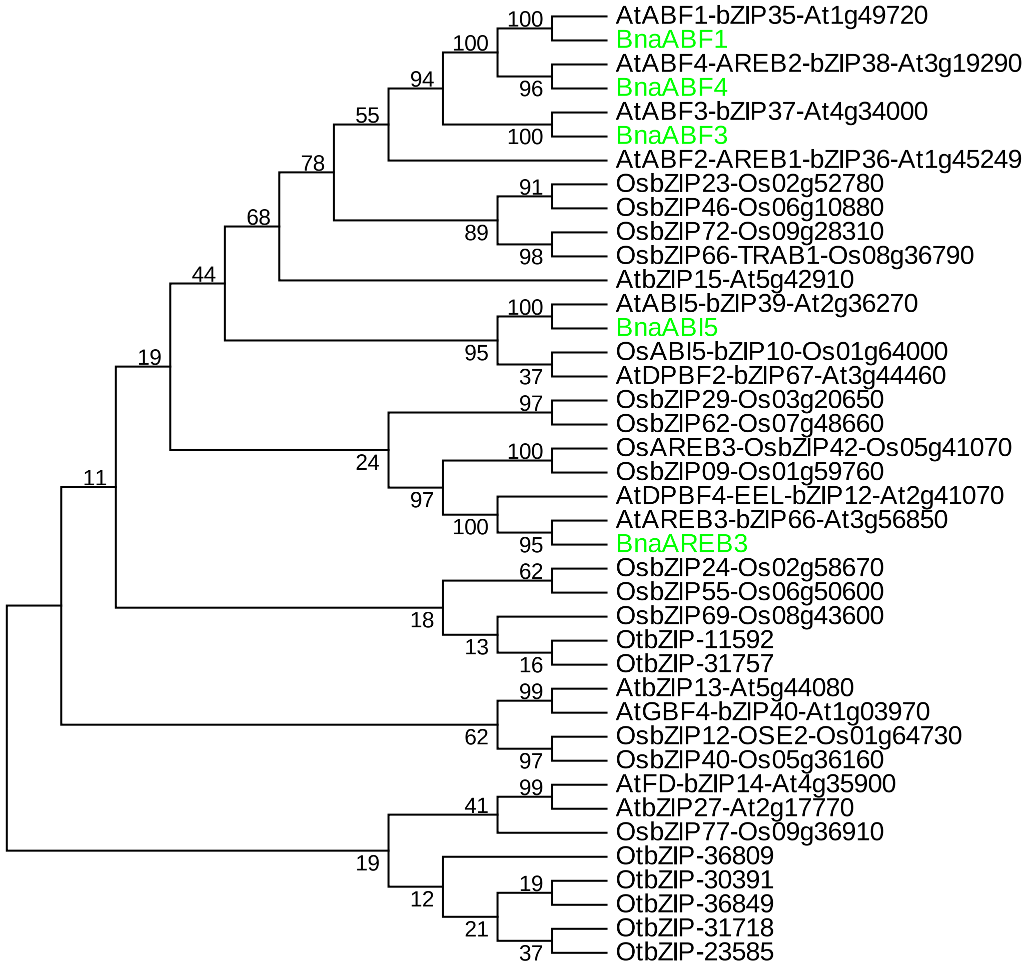
AtABF1-bZIP35-At1g49720
BnaABF1
AtABF4-AREB2-bZIP38-At3g19290
BnaABF4
AtABF3-bZIP37-At4g34000
BnaABF3
AtABF2-AREB1-bZIP36-At1g45249
OsbZIP23-Os02g52780
OsbZIP46-Os06g10880
OsbZIP72-Os09g28310
OsbZIP66-TRAB1-Os08g36790
AtbZIP15-At5g42910
AtABI5-bZIP39-At2g36270
BnaABI5
OsABI5-bZIP10-Os01g64000
AtDPBF2-bZIP67-At3g44460
OsbZIP29-Os03g20650
OsbZIP62-Os07g48660
OsAREB3-OsbZIP42-Os05g41070
OsbZIP09-Os01g59760
AtDPBF4-EEL-bZIP12-At2g41070
AtAREB3-bZIP66-At3g56850
BnaAREB3
OsbZIP24-Os02g58670
OsbZIP55-Os06g50600
OsbZIP69-Os08g43600
OtbZIP-11592
OtbZIP-31757
AtbZIP13-At5g44080
AtGBF4-bZIP40-At1g03970
OsbZIP12-OSE2-Os01g64730
OsbZIP40-Os05g36160
AtFD-bZIP14-At4g35900
AtbZIP27-At2g17770
OsbZIP77-Os09g36910
OtbZIP-36809
OtbZIP-30391
OtbZIP-36849
OtbZIP-31718
OtbZIP-23585

Figure S3. Multiple alignment, motif analysis and phylogenetic analysis of Clade A bZIP transcription factors in canola.

(A) Multiple alignment and motif analysis. Hyphens indicate gaps introduced to maximize the sequence alignment. Identical residues are highlighted in black, and similar residues are highlighted in gray. The basic region and leucine zipper repeat are shown by a thick line and dashed line under the sequences, respectively. The leucine (L) amino acid residues in the leucine zipper repeat are indicated by arrowheads. The multiple alignment was performed using the ClustalX1.83 and illustrated by BOXSHADE (http://www.ch.embnet.org/software/BOX_form.html).

(B) Phylogenetic analysis of canola Clade A bZIP proteins. Protein sequences were aligned using ClustalX (v1.83) and a maximum parsimony (MP) bootstrap consensus tree was drawn using MEGA5.1. The percentage of replicate trees is shown on the branches and it is calculated in the bootstrap test (1000 replicates) for the associated taxa being clustered together. Each taxon was named by a two to three letters representing the species followed by synonym and locus ID. The five BnabZIPs reported in this study are highlighted in green. The tree was rooted with bZIPs identified from the smallest free-living organism, *Ostreococcus tauri.* At, *Arabidopsis thaliana*; Bna, *Brassica napus*; Os, *Oryza sativa*; Ot, *Ostreococcus tauri.*
