# Supplementary material for: Identification, expression and interaction analyses of calcium-dependent protein kinase (CPK) genes in canola (Brassica napus L.)
Source: BMC Genomics. 2014 Mar 19;15:211. doi: 10.1186/1471-2164-15-211 (PMC4000008; doi:10.1186/1471-2164-15-211)
Supplement: Additional file 10: Figure S4 — Multiple alignment and phylogenetic analysis of Clade A PP2C proteins in canola. [file 1471-2164-15-211-S10.pdf]

(A)

```
BnaHAI2      1 MSDICYED--DALSCET-------------------------------------------
BnaHAI3      1 MADICYEVLTDASAYES-------------------------------------------
BnaAHG3      1 MAGICCGVVGESEPTVT-------------------------------------------
BnaAHG1      1 M-TEIYRTVSTGRKDDV-------------------------------------------
AtABI1       1 M-EEVSPAIAGP--------------------FRPFSETQMDFT----------------
BnaABI1      1 M-EEVSPAVAMP--------------------FMPFPEQQMELA----------------
BnaABI2      1 M-DKASPAVAAVP-------------------FRPFPDPQLEFA----------------
BnaHAB1      1 M-EEMTPAVAVTLSLANSICDS---SPVDITQLKNVTDAADLLP----------------
BnaHAB2      1 M-EEISPAVALTLSLANTMCDSGISSTLDITEMKNVADAVDMLSHQKDQSYSNGEVEHMM
consensus    1 * ........                           .

BnaHAI2     16 -------RPSWS--SRRRRIGVQRCRMS---------PSEMKQTPAVEDTEGIYKRNKQE
BnaHAI3     18 -------RPLHS--GRRQRFPMDKTVAM---------QEEWEKK--------NFKRNK--
BnaAHG3     18 -------VDSSTRASLRRRLDLLPSIKI---------VAPPLESSRKRQKRETPSGNQD-
BnaAHG1     17 -------SPTKCRERRRRRIEMRRQAAV---------FGEPSSSKNREETIYSGFVPLKK
AtABI1      24 -------GIRLGKGYCNNQYSNQDSENGDLMVSLPETSSCSVSGSHGSESRKVLISRINS
BnaABI1     24 -------GIMLGKGYCNGQYSSQDLENG----------SCSVSG-----SRKVLTSRINS
BnaABI2     25 -------GIR---GYCSPSLPESTCSSG--------------------EESTKDSSFIKI
BnaHAB1     41 ------DSTMEEEPKKGSCDGSVVDEDE-----VEDTSAVISEGLLVVDAGSELTLSMEI
BnaHAB2     60 EEDVSEDKTLTEARSLSSDIGVAVQESEEDEVLVSDDATIISEGLIVVNARSEVTLP-DT
consensus   61                    . .                          .     .  .

BnaHAI2     58 EYDFMNCASPPRSSAEGCSEGVESLLLDGEIRRDENNISGENSSVTGGVPYKKTVRETDA
BnaHAI3     50 --------------------------LEALTVRNEN-VSGESP-------------VTEA
BnaAHG3     61 --------------------------LESNVRSERNVEKKARSSPPVKNSNLIPSSATEA
BnaAHG1     61 HAR-----------------------TTTAAAEMGGLPADVGGVFPSPTSSHKKPEALVW
AtABI1      77 PNLNMKESAAAD-IVVVD--ISAGDEINGSD-------ITSEKKMISRTE--------SR
BnaABI1     62 PNLNMKKESPSS-SSSSSEIVVAGEEINGSD-------ERS-KKMISRTE--------SR
BnaABI2     55 NNMRQGSTSSSSRLADVTVDISAGEEINGSDEFDPRSTAQSEKRVLSRTE--------SR
BnaHAB1     90 DNGRVLAKAIILGESSIEEVPTAKVLIQDTKIEDCSGVTASEVVIRLPEENSNNHVAKGR
BnaHAB2    119 DNGRVLATAIIINETTIDQVPTAEVLITSLNHDVSMEVAASEVVIRLPEETHHNVARGSR
consensus  121  .  .                 .   . .           .   .    .         .

BnaHAI2    118 R-------PRYGVASVCGRRRDMEDTVAIHPSFVRKQ------------TEFSRARWHYF
BnaHAI3     70 S-------PRYGVSSVCGRRREMEDAVAIHPSFS----------------SHSEYPQHYF
BnaAHG3     95 ESCFVSDAPKIGTTSVCGRRRDMEDAVSVHHSLIHK----------------NSENLHFY
BnaAHG1     98 KGEEGDDEPMYGVVSVMGRSRKMEDTVTVKPSLCKP-------------EINQQKPVHFF
AtABI1     119 SLFEFKSVPLYGFTSICGRRPEMEDAVSTIPRFLQS--SSGSMLDGRFDP---QSAAHFF
BnaABI1    105 SLFEFKSVPLYGVTSICGRRPEMEDAVSTIPRFLQS--PTNSMLDGRFNP---QTTAHFF
BnaABI2    107 SLFEFKSVPLYGVTSICGRRPEMEDSVSAIPRFLQVSLLDCGRVANGLNP---HSSAHFF
BnaHAB1    150 SVYELDCIPLWGTVSIQGNRSEMEDAVAVLPHFLKLPIKMLMGDHEGMSPSLTHLTGHFF
BnaHAB2    179 SVYELECIPLWGTVSICGGRSEMEDAVTALPHCLKIPIKMLMGDHEGMSPSLTHLTSHFF
consensus  181 ....   .*..*..*..*.. .***.*........              .       *..
```

Catalytic domain

```
BnaHAI2    159 GIYDGHGCSHVASRCKERLHELVQEEAL---------ADKNEE---WKKMMERSFTRMDK
BnaHAI3    107 GVYDGHGCSHVAARCRERLHKLVQEELN---------SDREEEEDEWRKTMERSFTRMDK
BnaAHG3    139 GVFDGHGCSHVAEKCRERLHEIVKRDVE---------AMAAGGEDEWKETMAKSFQKMDR
BnaAHG1    145 AVYDGHGGSQVSTLCSTTMHTLVKEELEQ-PGCKLEGGGNDVVEEKWRGVMRRSFERMDE
AtABI1     174 GVYDGHGGSQVANYCRERMHLALAEEIAKEKPMLCDGDT---WLEKWKKALFNSFLRVDS
BnaABI1    160 GVYDGHGGSQVANYCRERMHLALAEEIAKEKPMLCDGDT---WQEKWKKALFNSFLRVDS
BnaABI2    164 GVYDGHGGSQVADYCRERMHLALTEEILKEKPEFCDGDT---WQEKWKRALFNSFMRVDF
BnaHAB1    210 GVYDGHGGYQVADYCRDRLHFALAEEIERIKDELCKRNTGEGRQVQWEKVFTSCFLNVDG
BnaHAB2    239 GVYDGHGGSQVADYCQDRIHFALAEEIERIKQELCERNTGEGRQVQWEKVFVDCYLKVDN
consensus  241 ...****...*. .*....* .. ... . .   ..  .     . *.. .  .....*
```

```
BnaHAI2    207 EAVRWEET-----VMSANCKCELQTPNCDAVGSTAVVSVITPEKIIVANCGDSRAVLCRN
BnaHAI3    158 EVVLLSES-----VVSAKCKCELQTPDCDAVGSTAVVSIITQDKIVVANCGDSRAVLCRN
BnaAHG3    190 EVSQRDSNSAASRSVKSSCRCELQSPQCDAVGSTAVVSVVTPEKIVVSNCGDSRAVLCRN
BnaAHG1    204 LAMGTCMR------RTTASLCHCDPREAAISGSTAVAAVLTNGNVVVANTGDSRAVLCRN
AtABI1     231 EIES--------------------VAPETVGSTSVVAVVFPSHIFVANCGDSRAVLCRG
BnaABI1    217 EVES--------------------VAPETVGSTSVVAVVFPTHIFVANCGDSRAVLCRG
BnaABI2    221 ELD---------------------FVPETVGSTSVVAVVFPTHIFVSNCGDSRAVLCRG
BnaHAB1    270 EIEGRIGRAA-AVVGGSSD-VVLEAVASETVGSTAVVALVCSSHLVVSNCGDSRAVLYRG
BnaHAB2    299 EVKGKISR---PVVGSSSDEMVLEAVSPETVGSTAVVALVCSSHIIVSNCGDSRAVLLRG
consensus  301 ..                     .     ...***.*.... .....*.*.*******.*.

BnaHAI2    262 GKPVPLSTDHKPDRPDELDRIQEAGGRVIYWDGPRVLGVLATSRAIGDNYLKPYVSSEPE
BnaHAI3    213 GKPFPLSTDHKPDRPDELDRIEGAGGRVIYWDCPRVLGVLAMSRAIGDNYLKPYVTCEPE
BnaAHG3    250 GVAIPLSSDHKPDRPDELIRIQQAGGRVIYWDGARVLGVLAMSRAIGDNYLKPYVIPDPE
BnaAHG1    258 GMAIPLSNDHKPDRPDERARIEAAGGRVLVVDGARVEGILATSRAIGDRYLKPMVAWEPE
AtABI1     270 KTALPLSVDHKPDREDEAARIEAAGGKVIQWNGARVFGVLAMSRSIGDRYLKPSIIPDPE
BnaABI1    256 KTALPLSTDHKPDREDEAARIEGAGGKVIRWNGARVFGVLAMSRSIGDRYLKPSIIPDPE
BnaABI2    259 KTPLALSVDHKPDREDEAARIEAAGGKVIQWNGARVFGVLAMSRSIGDKYLKPSVIPDPD
BnaHAB1    328 KEAMPLSVDHKPDREDEYARIENAGGKVIQWQGARVFGVLAMSRSLGDRYLKPYVIPEPE
BnaHAB2    356 KDSMPLSVDHKPDREDEYARIERAGGKVIQWQGARVSGVLAMSRSIGDEYLEPYVIPDPE
consensus  361 . ...** ******.** .**..***.*. ....** *.**.**..**.**.*.....*.

BnaHAI2    322 VTVTDRTEEDEFMILASDGLWDVVTNEAACATVQMYLNKKGGRGGRRREATECEERKEE
BnaHAI3    273 VTVTDRT-DDDCLILASDGLWDVVSNETACSVARMCLS------GGRRRRGTP------E
BnaAHG3    310 VTVTDRTDDDECLILASDGLWDVVTNETACGVARMCLQGAAAADGGDSDTAHN-------
BnaAHG1    318 VTFMRREPGDECLILASDGLWDVLSSQLACDIARFCLREDAPSGLDLNETATEDDNEGQG
AtABI1     330 VTAVKRVKEDDCLILASDGVWDVMTDEEACEMARKRILLWHKKNAVAGDASLLADERRKE
BnaABI1    316 VTAVRRVKEDDCLILASDGVWDVMTDEEACEMARKRILLWHKKNAVAGDASLHTDERRGE
BnaABI2    319 VTSIRRVKEDDCLILASDGLWDVMTNEEVCDMARKRILLWHKKNAMAGDALLPAE-KRGE
BnaHAB1    388 VTFMPRSREDDCLIIASDGLWDVMSNQEVCEVARKRILMWHKKHGAP------PLAERGK
BnaHAB2    416 VTFMPRAREDECLILASDGLWDVISNQDACEFARKRILWWHKKNGAL------PLAERGV
consensus  421 ** . * ..*...*.****.***......*........ .... ..  .      .  . .

BnaHAI2    382 KAVGSRKNGKRGEITDKACTEASVLLTKLALAKHSCDNVSVVVIELSRRRKRHVA---
BnaHAI3    320 QAVET--------ISDKACMEASVLLTKLALARHSGDNVSVVVIDLRRKGHVTGH---
BnaAHG3    363 -----------------ACSDASLLLTKLALARQSSDNVSVVVDLRKRRSNNQVS--
BnaAHG1    378 SSGQN----------PSRSVLAATLLTRLALGRQSNDNISVIVIDLMNSPP-------
AtABI1     390 GK-------------DPAAMSAAEYLSKLAIQRGSKDNISVVVVDLKPRRKLKSKPLN
BnaABI1    376 GK-------------DPAAMSAAEYLSKLALQRGSKDNITVVVVDLKPQRKFKSKPLN
BnaABI2    378 GK-------------DPAAMSAAEYLSKMALQRGSKDNISVIVVDLKGIRKFKSKALN
BnaHAB1    442 GT-------------DPACQAAAEYLSVLALQKGSKDNISIIVVDLKAQRKFKTRA--
BnaHAB2    470 GE-------------DQACQAASDYLSKLAIQKGSKDNISIIVVDLKAQRKFKTKS--
consensus  481 .                . ... *...*...*....*.**....*..*.  .. ...
```

(B)

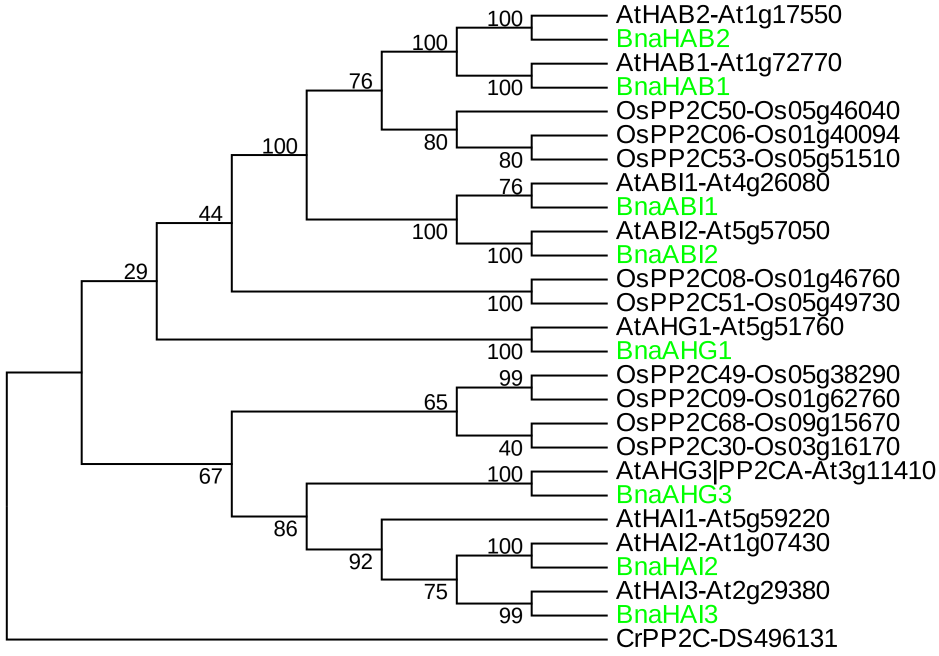

Figure S4. Multiple alignment and phylogenetic analysis of Clade A PP2C proteins in canola. (A) Multiple alignment of Clade A PP2C proteins in canola. Hyphens indicate gaps introduced to maximize the sequence alignment. Identical residues are highlighted in black, and similar residues are highlighted in gray. The catalytic domain is shown by a thick line under the sequences. The multiple alignment was performed using the ClustalX1.83 and illustrated by BOXSHADE (http://www.ch.embnet.org/software/BOX_form.html).

(B) Phylogenetic analysis of canola Clade A PP2C proteins. Protein sequences were aligned using ClustalX (v1.83) and a maximum parsimony (MP) bootstrap consensus tree was drawn using MEGA5.1. The percentage of replicate trees is shown on the branches and it is calculated in the bootstrap test (1000 replicates) for the associated taxa being clustered together. Each taxon was named by a two to three letters representing the species followed by synonym and locus ID. The eight BnaPP2Cs reported in this study are highlighted in green. The tree was rooted with a PP2C identified from the unicellular green algae, *Chlamydomonas reinhardtii*. At, *Arabidopsis thaliana*; Bna, *Brassica napus*; Os, *Oryza sativa*; Cr, *Chlamydomonas reinhardtii*.
